# *Orientia tsutsugamushi* Stimulates an Original Gene Expression Program in Monocytes: Relationship with Gene Expression in Patients with Scrub Typhus

**DOI:** 10.1371/journal.pntd.0001028

**Published:** 2011-05-17

**Authors:** Wiwit Tantibhedhyangkul, Thanavadee Prachason, Duangdao Waywa, Adil El Filali, Eric Ghigo, Wanna Thongnoppakhun, Didier Raoult, Yupin Suputtamongkol, Christian Capo, Chanin Limwongse, Jean-Louis Mege

**Affiliations:** 1 Unité de Recherche sur les Maladies Infectieuses Tropicales et Emergentes, Centre National de la Recherche Scientifique - Institut de Recherche pour le Développement Unité Mixte de Recherche 6236, Université de la Méditerranée, Faculté de Médecine, Marseille, France; 2 Division of Molecular Genetics, Department of Research and Development, Mahidol University, Bangkok, Thailand; 3 Department of Immunology, Mahidol University, Bangkok, Thailand; 4 Department of Medicine, Faculty of Medicine Siriraj Hospital, Mahidol University, Bangkok, Thailand; University of Texas Medical Branch, United States of America

## Abstract

*Orientia tsutsugamushi* is the causal agent of scrub typhus, a public health problem in the Asia-Pacific region and a life-threatening disease. *O. tsutsugamushi* is an obligate intracellular bacterium that mainly infects endothelial cells. We demonstrated here that *O. tsutsugamushi* also replicated in monocytes isolated from healthy donors. In addition, *O. tsutsugamushi* altered the expression of more than 4,500 genes, as demonstrated by microarray analysis. The expression of type I interferon, interferon-stimulated genes and genes associated with the M1 polarization of macrophages was significantly upregulated. *O. tsutsugamushi* also induced the expression of apoptosis-related genes and promoted cell death in a small percentage of monocytes. Live organisms were indispensable to the type I interferon response and apoptosis and enhanced the expression of M1-associated cytokines. These data were related to the transcriptional changes detected in mononuclear cells isolated from patients with scrub typhus. Here, the microarray analyses revealed the upregulation of 613 genes, which included interferon-related genes, and some features of M1 polarization were observed in these patients, similar to what was observed in *O. tsutsugamushi*-stimulated monocytes in vitro. This is the first report demonstrating that monocytes are clearly polarized in vitro and ex vivo following exposure to *O. tsutsugamushi*. These results would improve our understanding of the pathogenesis of scrub typhus, during which interferon-mediated activation of monocytes and their subsequent polarization into an M1 phenotype appear critical. This study may give us a clue of new tools for the diagnosis of patients with scrub typhus.

## Author Summary

Scrub typhus, a life-threatening disease that occurs in the Asia-Pacific region, is a public health problem since a billion of people are at risk and one million new cases arise each year. *Orientia tsutsugamushi*, the causal agent of scrub typhus, is an obligate intracellular bacterium that mainly infects endothelial cells. We demonstrated here that *O. tsutsugamushi* grew in monocytes isolated from healthy donors and altered the expression of a large number of genes including interferon-related genes, genes associated with the M1 polarization of macrophages and apoptosis-related genes. Importantly, these data were related to the transcriptional changes detected in mononuclear cells isolated from patients with scrub typhus. Indeed, the microarray analyses revealed the upregulation of numerous genes, which included interferon-related genes, and some features of M1 polarization. This is the first report demonstrating that monocytes are clearly polarized in vitro and ex vivo following exposure to *O. tsutsugamushi*. These results improve our understanding of the pathogenesis of scrub typhus and may give us a clue of new tools for the diagnosis of patients with scrub typhus.

## Introduction

*Orientia tsutsugamushi* is the causative agent of scrub typhus, a life-threatening disease characterized by fever, lymphadenopathy, rash and eschar that can be complicated by interstitial pneumonitis, meningitis and myocarditis [1]. The proper diagnosis of scrub typhus can be difficult due to the non-specific initial symptoms that are frequently found in other acute febrile illnesses. While scrub typhus is confined geographically to the Asia-Pacific region, a billion of people are at risk and one million new cases arise each year. As *O. tsutsugamushi* is transmitted to humans by the bite of larval trombiculid mites, people who inhabit regions infested with these vectors are at high risk for acquiring scrub typhus [2]. To date, no effective strategy has succeeded in generating long lasting, protective immunity to this particular infection despite aggressive attempts to develop a prophylactic vaccine [3].

Due to the significant genetic and phenotypic differences in its cell wall, including the absence of peptidoglycan and lipopolysaccharide (LPS), *O. tsutsugamushi* has been classified as a new genus that is distinct from the *Rickettsia* genus [4]. The complete genome sequence of two *O. tsutsugamushi* strains (Boryong and Ikeda) has recently been described. The *O. tsutsugamushi* genome contains several repetitive sequences, including genes for conjugative type IV secretion systems (*tra* genes) [5], [6]. *O. tsutsugamushi* is an obligate intracellular bacterium that can invade a variety of cell types both in vitro and in vivo. It has been recently shown that *O. tsutsugamushi* can exploit α5β1 integrin-mediated signaling and the actin cytoskeleton to invade HeLa cells [7]. Another study reported that following phagocytosis by L929 cells, *O. tsutsugamushi* rapidly escapes the phagosome and enters the cytosol [1]. *O. tsutsugamushi* also infects endothelial and fibroblast cell lines through clathrin-mediated endocytosis [8]. Once inside the cell, *O. tsutsugamushi* moves along microtubules to the microtubule-organizing center in a dynein-dependent manner [9]. In experimental animals, *O. tsutsugamushi* infects peritoneal mesothelial cells [10], macrophages [11] and polymorphonuclear leukocytes [12]. In humans, *O. tsutsugamushi* has been detected in peripheral blood mononuclear cells (PBMCs) from patients with acute scrub typhus [13].

The mechanisms governing the interaction between *O. tsutsugamushi* and host cells are only partially understood. It has been recently demonstrated that the expression of approximately 30% of bacterial genes is modulated when *O. tsutsugamushi* is cultured in eukaryotic cells. When compared to the bacterial gene expression seen in the L929 fibroblast cell line, the expression of a number of bacterial genes involved in translation, protein folding and secretion is downregulated in J774 macrophages, and this decreased expression correlated with the reduced growth of *O. tsutsugamushi* in macrophages [14]. Infection with *O. tsutsugamushi* most likely has many effects on the human immune response. In vitro studies have shown that *O. tsutsugamushi* induces the expression of genes encoding chemokines, including MCP-1 (CCL2), RANTES (CCL5) and IL-8 (CXCL8), in human endothelial cells [15], [16]. In patients with scrub typhus, the serum level of pro-inflammatory cytokines (e.g. TNF, IL-12p40, IL-15, IL-18 and IFN-γ) is also increased [17], demonstrating that *O. tsutsugamushi* infection is accompanied by an inflammatory response [18]. The circulating levels of chemokines such as CXCL9 (MIG) and CXCL10 (IP-10), which are known to attract Th1, cytotoxic T cells and NK cells, and molecules such as granzymes A or B, which are released following the degranulation of cytotoxic lymphocytes, are also increased [18].

In this paper, we report that *O. tsutsugamushi* induces large changes in gene transcription in naïve human monocytes. In addition to genes encoding inflammatory cytokines and chemokines, *O. tsutsugamushi* upregulates the expression of genes involved in type I IFN pathway and genes involved in apoptosis. Interestingly, these in vitro results were related to the expression of genes involved in the immune response, including the IFN response, in patients with scrub typhus. Our study highlights the role of IFN-mediated monocyte activation in the pathogenesis of scrub typhus.

## Materials and Methods

### Ethics Statement

Blood samples from patients and controls were collected after informed and written consent obtained from each participant, and the study was conducted with the approval of the Ethics Committee of Siriraj Hospital, Bangkok, Thailand.

### Patients

Ten milliliters of blood was collected from patients with acute undifferentiated fever who were seen at Siriraj Hospital or Ban Mai Chaiyapot Hospital. The clinical status of each patient was recorded. Within two hours of blood drawing, PBMCs were separated by Ficoll density gradient centrifugation. The PBMCs were immediately lysed in Trizol reagent (Invitrogen, Carlsbad, CA), as recommended by the manufacturer, and the lysates were stored at −80°C until further analysis. The study participants were retrospectively divided into the following three groups: healthy controls (individuals without any of the four infections), patients with scrub typhus (n = 4) and an infected control group consisting of patients with murine typhus (n = 7), malaria (n = 4) or dengue (n = 7). Patients with evidence suggesting co-infections or those with malignancies were excluded. The diagnostic criteria for scrub typhus were the presence of circulating *O. tsutsugamushi*-specific IgM with a titer greater than 1∶400 in serum from patients with acute disease and/or *O. tsutsugamushi*-specific IgG with at least a four-fold increase of titer. The criteria for murine typhus were a serum *Rickettsia typhi*-specific IgM titer greater than 1∶400 in patients with acute disease and/or at least a four-fold increase of IgG titer. The criteria for dengue virus infection were a dengue virus-specific IgM titer greater than 1∶1,280, as determined using enzyme linked immunosorbent assay (ELISA), and/or positive for dengue RNA using RT-PCR. Malaria infection was determined by the detection of *Plasmodium* species in blood films observed using a light microscope. Patients with murine typhus, malaria or dengue presented with a lower absolute number of leukocytes than patients with scrub typhus, but the lymphocyte/monocyte ratio was similar (Table S1). Ten healthy individuals were included in the study as controls, and the extracted RNA was pooled to reduce the effect of interindividual variability. The reproducibility of this procedure was tested using two different pools consisting of 5 individuals each.

### *O. tsutsugamushi* culture and isolation

*O. tsutsugamushi*, strain Kato (CSUR R163), was propagated in L929 cells cultured in minimum essential media (MEM) supplemented with 5% fetal bovine serum (FBS) and 2 mM L-glutamine (Invitrogen, Cergy Pontoise, France), as recently described [19]. When almost 100% of the cells were infected, as determined using May Grünwald Giemsa (Merck, Darmstadt, Germany) staining, the cells were harvested, lysed using glass beads and centrifuged at 500× *g* for 5 min to remove cell debris. The supernatants were centrifuged at 2,000× *g* for 10 minutes to collect bacterial pellets. The isolated bacteria were frozen in MEM containing 20% FBS and 5% DMSO until use. The titer of the supernatants was determined as described previously [8], [20] with slight modifications. Briefly, the bacteria were serially diluted five-fold and incubated with L929 cells grown in 24-well plates. After 2 hours, free bacteria were removed and the infected L929 cells were cultured in MEM containing 5% FBS and 0.4 µg/ml daunorubicin (Biomol, Hamburg, Germany), which partially inhibits cell growth, for 2 days [21]. The infection of the L929 cells was quantified using indirect immunofluorescence with pooled serum from Thai patients with scrub typhus at a dilution of 1∶400 and fluorescein isothiocyanate-conjugated goat anti-human IgG (BioMérieux, Marcy l'Etoile, France) diluted at 1∶200 as a secondary antibody. The infected-cell counting units (ICUs) of *O. tsutsugamushi* were defined as (the total number of cells used in the infection)×(the percentage of infected cells)×(the dilution rate of the bacterial suspension)/100. In some experiments, *O. tsutsugamushi* organisms were killed by heating at 100°C for 5 minutes.

### Infection of human monocytes

PBMCs were isolated from leukopacks (Etablissement Français du Sang, Marseille, France) over a Ficoll gradient (MSL, Eurobio, Les Ulis, France) and incubated in 24-well plates for 1 hour. Adherent cells were designed as monocytes since more than 90% of them expressed CD14, as previously described [22]. Monocytes (1.5×10^5^ per assay) were incubated with 3×10^5^ *O. tsutsugamushi* organisms in RPMI 1640 containing 10% FBS, 20 mM HEPES and 2 mM L-glutamine (Invitrogen) for 2 hours. The monocytes were then extensively washed to remove free organisms and cultured for the indicated times. The uptake and the intracellular fate of the *O. tsutsugamushi* organisms were determined using immunofluorescence and quantitative real-time PCR (qPCR). Immunofluorescence was performed using pooled serum from Thai patients with scrub typhus and a standard protocole. Cells were then examined by fluorescence and laser scanning microscopy using a confocal microscope (Leica TCS SP5, Heidelberg, Germany) as recently described [23].

To assess bacterial DNA, the monocytes were incubated in 0.1% Triton X-100, and DNA was extracted in a 150 µl volume using a QIAamp Tissue Kit (Qiagen, Courtaboeuf, France), as recommended by the manufacturer. The number of bacterial DNA copies was calculated using the Taqman system (Applied Biosystems, Warrington, UK) with a 5 µl DNA sample. The selected primers and probes were designed based on the available DNA sequence of *O. tsutsugamushi* strain Boryong (complete genome, GenBank ref. NC_009488.1) and were the following: forward (3235–3257), 5′-AAGCATAGGTTACAGCCTGGWGA-3′; reverse (3346–3373), 5′-ACCCCAACGGATTTAATACTATATCWAC-3′; probe R (3307–3338), 5′-FAM-CCATCTTCAAGAAATGGCATATCTTCCTCAGG- TAMRA-3′. The resulting PCR product was 139 bp in size. Negative controls consisted of DNA extracted from uninfected monocytes. Each PCR run included a standard curve generated from tenfold serial dilutions of a known concentration of *O. tsutsugamushi* DNA. The results are expressed as the total number of bacterial DNA copies.

### Transcriptional profile of monocytes

Eight hours post-infection, total RNA was extracted from infected and uninfected monocytes using an RNeasy Mini kit (Qiagen) with DNase digestion, as recommended by the manufacturer. The quality of the isolated RNA was assessed by Agilent 2100 Bioanalyzer and an RNA 6000 Nano Kit (Agilent Technologies, Massy, France). The concentration of RNA was determined using a NanoDrop 1000 spectrophotometer (Thermo Scientific, Wilmington, DE, USA). A total of eight RNA samples (four samples per condition) were then processed for microarray analysis. The RNA was amplified and Cy3-labeled cDNA was generated using the Agilent Low RNA Input Fluorescent Linear Amplification Kit (Agilent Technologies), as recommended by the manufacturer. The amplified cDNA was processed and hybridized to 4×44K microarray slides (Agilent Technologies). The scanned images were analyzed with Feature Extraction Software 10.5.1 (Agilent) using default parameters. The data processing and analyses were performed using the Resolver software 7.1 (Rosetta Inpharmatics, Cambridge, MA) as previously described [24] and R and BioConductor softwares. The Rosetta intensity error model for single color microarrays was used to perform inter-array normalization. Statistical analyses were performed using the significance analysis of microarrays (SAM) [25]. The median false discovery rate was approximately 5%. Genes with an absolute fold change (FC) greater than 2 and a *p* value of the error model less than 0.01 were considered differentially modulated. The differentially expressed genes were classified based on their Gene Ontology (GO) category. The transcriptional profile of the infected monocytes was compared with the profile of monocytes stimulated with IFN-γ or IL-4 to assess their polarization status (manuscript in preparation) using R software and hierarchical clustering.

Minimum Information About a Microarray Experiment (MIAME)-compliant data sets are provided in the Gene Expression Omnibus (GEO) [26] at the National Center for Biotechnology Information (http://www.ncbi.nlm.nih.gov/geo/) and can be assessed through GEO series accession number GSE24247 (http://www.ncbi.nlm.nih.gov/geo/query/acc.cgi?acc=GSE24247).

### Transcriptional profile of patient PBMCs

The RNA from PBMCs lysed in TRIZOL was extracted using the RNeasy Mini Kit (Qiagen), and the RNA of ten healthy individuals was divided into two distinct pools. The RNA amplification for microarray analysis was performed using the Illumina TotalPrep RNA Amplification Kit (Ambion, Austin, TX), as recommended by the manufacturer. Five hundred nanograms of amplified cRNA was hybridized onto Human-6 v2 BeadChips (Illumina, San Diego, CA), which contained more than 46,000 probes targeting all known human transcripts. The hybridized chips were scanned on an Illumina BeadStation 500 and assessed for fluorescent signal intensity using Illumina Beadstudio software. Normalization and all analyses of microarray data were performed by GeneSpring GX 9 demo version (Agilent Technologies). Briefly, quantile normalization was applied to the raw signal intensities. Next, the probes in which the normalized expression level was below the twentieth percentile for every sample in any group of patients were excluded, leaving 38,630 probes for further analysis. We focused on two main sets of genes. The first one comprised scrub typhus-associated genes, which were identified by performing a Welch ANOVA with Benjamini-Hochberg correction [27] across the four disease groups. A post hoc Tukey's HSD test was further applied to the Welch ANOVA results. Genes that were differentially expressed in scrub typhus patients compared to the other groups were then selected using the intersection rule. Unsupervised hierarchical cluster was performed for all patient groups on the basis of the Euclidean distance and average linkage. The second gene set was composed of scrub typhus-responsive genes, which were genes whose mean expression level in patients with scrub typhus group was at least twofold greater than the expression in healthy controls. The significance of the GO enrichment was evaluated using the hypergeometric formula with Benjamini-Yekutieli correction [27], [28]. As the transcriptomic profiles of patient PBMCs and those of naïve monocytes stimulated with *O. tsutsugamushi* were obtained using Illumina bead chips and Agilent microarrays, respectively, the two profiles were compared by building a virtual Agilent microarray. These genes were then analyzed using R software. The data are generated in compliance with the MIAME guidelines and have been deposited in the NCBI's Gene Expression Omnibus and are accessible using GEO Series accession number GSE16463 (http://www.ncbi.nlm.nih.gov/geo/query/acc.cgi?token=bxmzrwwgygmwahu&acc=GSE16463).

### Quantitative real time RT-PCR and ELISA

Real time quantitative RT-PCR (qRT-PCR) of the genes of interest was carried out as previously described [24]. Briefly, total RNA was isolated from monocytes using a Qiagen kit, and cDNA synthesis was performed using an oligo(dT) primer and M-MLV reverse transcriptase (Invitrogen), as recommended by the manufacturer. Real time PCR from cDNA templates was performed using Light Cycler-FastStart DNA Master^PLUS^ SYBR Green I (Roche Applied Science, Meylan, France). The sequences of the primers are provided in Table S2. The fold change of the target genes relative to β-actin was calculated using the 2^−ΔΔCt^ method, as described previously [29]. The level of IFN-β and TNF in the supernatants was analyzed by IFN-β and TNF ELISA kits (R&D Systems, Lille, France). The IL-1β level was determined using an IL-1β ELISA kit purchased from Diaclone (Besançon, France).

### *In situ* cell death

Infected monocytes were fixed with 3% paraformaldehyde before being analyzed using the TdT-mediated dUTP nick-end labeling (TUNEL) assay. DNA strand breaks were labeled using an In Situ Cell Death Detection Kit, TMR red (Roche Applied Science), as recommended by the manufacturer. Nuclei were counter-stained with DAPI. The number of TUNEL-positive cells and DAPI-stained nuclei was determined using fluorescence microscopy. Cell death is expressed as the ratio of TUNEL-positive cells to DAPI-stained nuclei ×100.

### Statistical analyses

Statistical analyses were performed using GraphPad Prism Sofware v. 5.01. The results are expressed as the mean ± SEM and were compared using the non-parametric Mann-Whitney *U* test: *p* values less than 0.05 were considered significant.

## Results

### *O. tsutsugamushi* replicates in human monocytes

**Figure 1:**
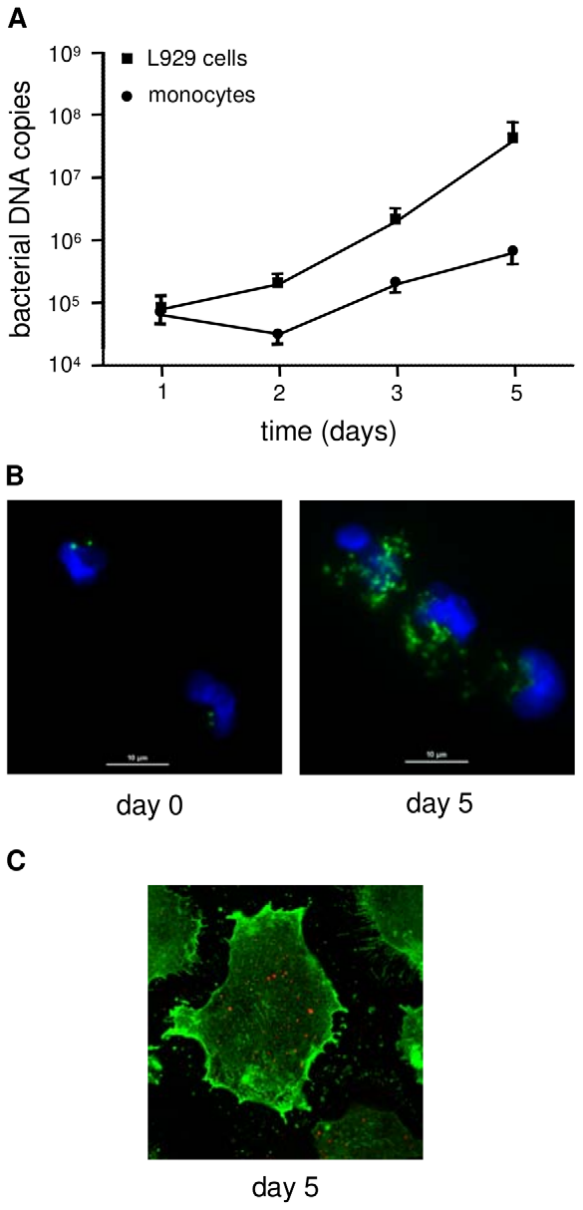
*O. tsutsugamushi* replication within monocytes. Monocytes and L929 cells were infected with *O. tsutsugamushi* (two viable bacteria per cell) for different periods of time. A. The number of bacterial DNA copies was determined using qRT-PCR. The data are expressed as the mean ± SD of two independent experiments performed in triplicate. B. Monocytes were infected with *O. tsutsugamushi*, and the bacteria were detected using indirect immunofluorescence. C. Monocytes infected with *O. tsutsugamushi* for 5 days were labeled with bodipy phallacidin to detect filamentous actin and bacteria were detected using indirect immunofluorescence (in red). One representative micrograph performed in confocal microscopy is shown.

As *O. tsutsugamushi* organisms have been detected in monocytes from patients with acute scrub typhus [13], we investigated whether they were able to invade and replicate within naïve monocytes. Monocytes were incubated with *O. tsutsugamushi* (two bacteria per cell) for 2 hours, extensively washed to remove free organisms and incubated for the indicated times. After 1 day, the number of bacterial DNA copies was approximately 1×10^5^ (Figure 1A). Using immunofluorescence, the uptake of *O. tsutsugamushi* by monocytes could be readily observed (Figure 1B). The number of bacterial DNA copies steadily increased over the course of the 5-day experiment (Figure 1A). The replication of *O. tsutsugamushi* in monocytes was slower than in L929 cells (Figure 1A), a permissive control cell line. Indeed, the doubling time of *O. tsutsugamushi* within monocytes was approximately 18 hours compared with 10 hours in L929 cells. Time-dependent replication of *O. tsutsugamushi* within monocytes was also detected using indirect immunofluorescence (Figure 1B) and confocal microscopy (Figure 1C). These data demonstrate that *O. tsutsugamushi* was capable of replication in naïve monocytes.

### Global transcriptome analysis of *O. tsutsugamushi*-infected monocytes

To understand how *O. tsutsugamushi* inhibits the microbicidal machinery of monocytes, we compared the transcriptional profile of unstimulated monocytes to that of monocytes stimulated with *O. tsutsugamushi* for 8 hours using whole genome microarrays.

**Figure 2:**
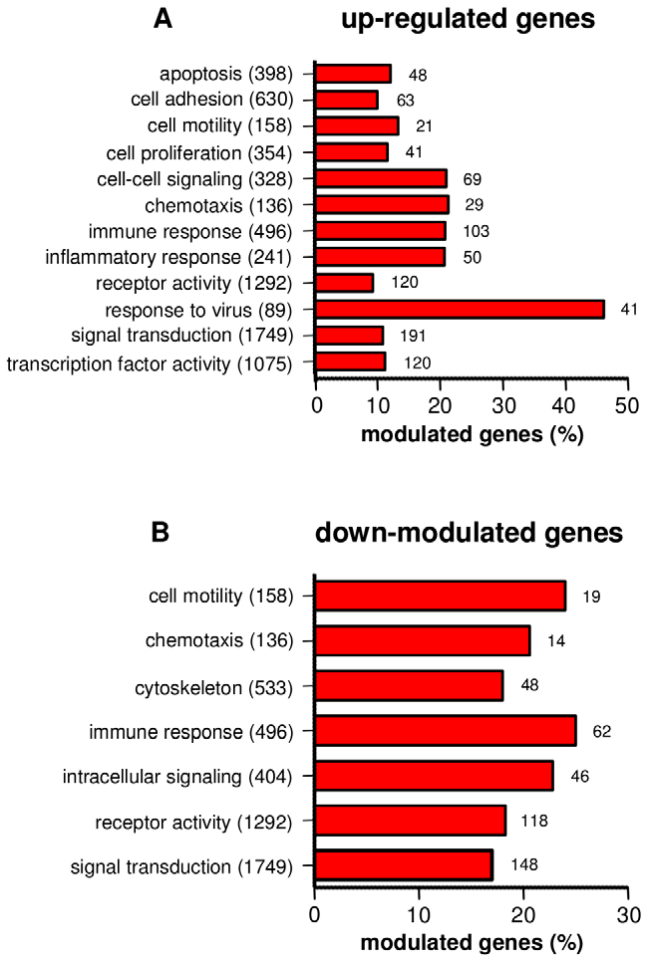
GO analysis of differentially expressed genes. Monocytes were stimulated with *O. tsutsugamushi* or mock stimulated for 8 hours, and the modulation of genes was analyzed using microarrays and GO term tools. The upregulated (A) and downregulated (B) genes were classified based on the major biological processes in which they are involved. The total number of genes present in each biological process and the number of differentially expressed genes are indicated. The results are expressed as the percentage of the upregulated or downregulated genes.

This time was determined as follows. The time course of the expression of genes encoding cytokines (TNF, IL-1β, IL-6, IL-12p40), chemokines (CXCL10, CXCL11), IFN-β and TNF-related apoptosis-inducing ligand (TRAIL/Apo2L/TNFSF10) was studied using qRT-PCR. Their expression was variable after 2 hours, was maximum after 8 hours and progressively decreased thereafter (Figure S1). Consequently, the time of 8 hours was used to stimulate monocytes with *O. tsutsugamushi*. We found that 4,762 genes were altered in response to *O. tsutsugamushi*: 2,380 genes were upregulated and 2,382 genes were downregulated with at least a twofold change and a *p* value less than 0.01. The differentially expressed genes were classified into different categories based on their GO according to their *p* value. Among the upregulated genes (2–100-fold increased) with an enrichment higher than 20% were genes involved in the immune response, the inflammatory response, chemotaxis, the anti-viral response and cell-cell signaling (Figure 2A). Other GO categories related to cellular processes, including apoptosis, cell proliferation, cell-cell signaling, receptor activity, signal transduction and transcription factor activity, exhibited lower enrichments, ranging from 10 to 20% (Figure 2A). Among the downregulated genes were genes involved in cell motility, chemotaxis, the cytoskeleton, the immune response, intracellular signaling, receptor activity and signal transduction (Figure 2B). Taken together, these data demonstrate that *O. tsutsugamushi* activated an important transcriptional program in human monocytes.

### Analysis of the *O. tsutsugamushi*-induced type I IFN signature

**Figure 3:**
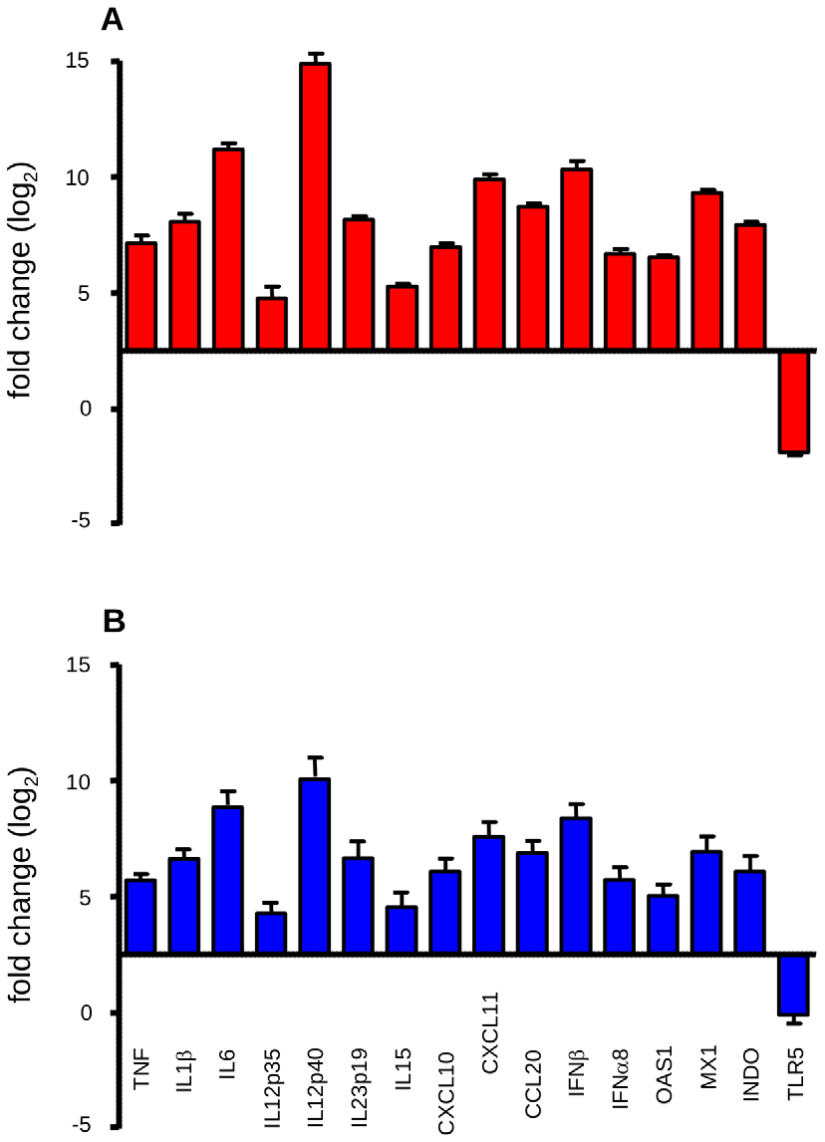
Quantitative RT-PCR of selected genes in stimulated monocytes. Monocytes were stimulated with or without *O. tsutsugamushi* for 8 (A) and 24 (B) hours. RNA was extracted, and qRT-PCR was performed on 16 genes involved in the immune response that were differentially expressing in the microarray experiments. The results, expressed as the log_2_ ratio of fold changes, are presented as the mean ± SEM of three experiments performed in triplicate.

As the GO category “response to virus” was specifically enriched following exposure to *O. tsutsugamushi*, we investigated the type I IFN pathway in monocytes. The genes encoding IFN-β, four subtypes of IFN-α and interferon-stimulated genes (ISGs) were upregulated in monocytes infected with *O. tsutsugamushi* (Table 1). These upregulated ISGs included the genes encoding CCL8, CXCL10 (IP10), CXCL11 (I-TAC), 2′5′ oligoadenylate synthetase (OAS 1–3, OASL), myxovirus resistance (MX1 and MX2), ISG15, ISG20, interferon regulatory factor 7 (IRF7), many interferon-induced proteins, the promyelocytic leukemia gene product (PML) and some metallothioneins. The upregulation of a type I IFN signature (the genes encoding IFN-β, IFN-α8, OAS1 and MX1) was confirmed using qRT-PCR in monocytes from three distinct donors incubated with *O. tsutsugamushi* for 8 (Figure 3A) and 24 hours (Figure 3B). Monocytes were also incubated with heat-killed *O. tsutsugamushi* for 8 hours, and the mRNA expression level of IFN-β and ISGs (MX1, CXCL10 and CXCL11) was determined using qRT-PCR. In contrast to live *O. tsutsugamushi*, heat-killed cells failed to induce the expression of IFN-β and ISGs (MX1, CXCL10 and CXCL11) in monocytes. We then investigated whether the *O. tsutsugamushi*-induced type I IFN signature was functional. Monocytes were incubated with *O. tsutsugamushi* for 24 hours, and the secretion of IFN-β was determined using ELISA. Monocytes infected with live *O. tsutsugamushi* produced 65±16 pg/ml IFN-β whereas those incubated with heat-killed bacteria were unable to produce IFN-β. These data clearly demonstrate that only live *O. tsutsugamushi* induced a sustained type I IFN response in human monocytes.

**Table 1:** Modulated genes in the “response to virus” GO term.

Gene function and full gene name	Gene symbol	GenBank ID	Fold change
apolipoprotein B mRNA editing enzyme, catalytic polypeptide-like 3G	APOBEC3G	NM_021822	3.1
chemokine (C-C motif) ligand 4	CCL4	NM_002984	16.9
chemokine (C-C motif) ligand 8	CCL8	NM_005623	74.8
DEAD (Asp-Glu-Ala-Asp) box polypeptide 58	DDX58	NM_014314	36.8
eukaryotic translation initiation factor 2-alpha kinase 2	EIF2AK2	NM_002759	13.0
interferon, gamma-inducible protein 16	IFI16	NM_005531	3.5
interferon-induced protein 35	IFI35	NM_005533	7.6
interferon-induced protein 44	IFI44	NM_006417	29.4
interferon induced with helicase C domain 1	IFIH1	NM_022168	18.0
interferon, alpha 4	IFNA4	NM_021068	58.8
interferon, alpha 5	IFNA5	NM_002169	60.5
interferon, alpha 6	IFNA6	NM_021002	7.6
interferon, alpha 8	IFNA8	NM_002170	71.7
interferon, beta 1, fibroblast	IFNB1	NM_002176	71.7
interferon, gamma	IFNG	NM_000619	5.1
interleukin 23, alpha subunit p19	IL23A	NM_016584	5.1
interleukin 28 receptor, alpha (interferon, lambda receptor)	IL28RA	NM_170743	2.3
interleukin 29 (interferon, lambda 1)	IL29	NM_172140	5.7
interferon regulatory factor 7	IRF7	NM_004031	14.9
ISG15 ubiquitin-like modifier	ISG15	NM_005101	54.1
interferon stimulated exonuclease gene 20 kDa	ISG20	NM_002201	32.4
myxovirus (influenza virus) resistance 1	MX1	NM_002462	26.7
myxovirus (influenza virus) resistance 2	MX2	NM_002463	50.2
2′,5′-oligoadenylate synthetase 1, 40/46 kDa	OAS1	NM_002534	13.6
phospholipid scramblase 1	PLSCR1	NM_021105	5.0
v-rel reticuloendotheliosis viral oncogene homolog A	RELA	BC014095	3.1
signal transducer and activator of transcription 2, 113 kDa	STAT2	NM_005419	5.9
TANK-binding kinase 1	TBK1	NM_013254	2.0
tumor necrosis factor (TNF superfamily, member 2)	TNF	NM_000594	13.9
tripartite motif-containing 22	TRIM22	NM_006074	8.4
tripartite motif-containing 5	TRIM5	NM_033034	10.7
tripartite motif-containing 5	TRIM5	NM_033092	13.6

### Analysis of the inflammatory response induced by *O. tsutsugamushi*

**Figure 4:**
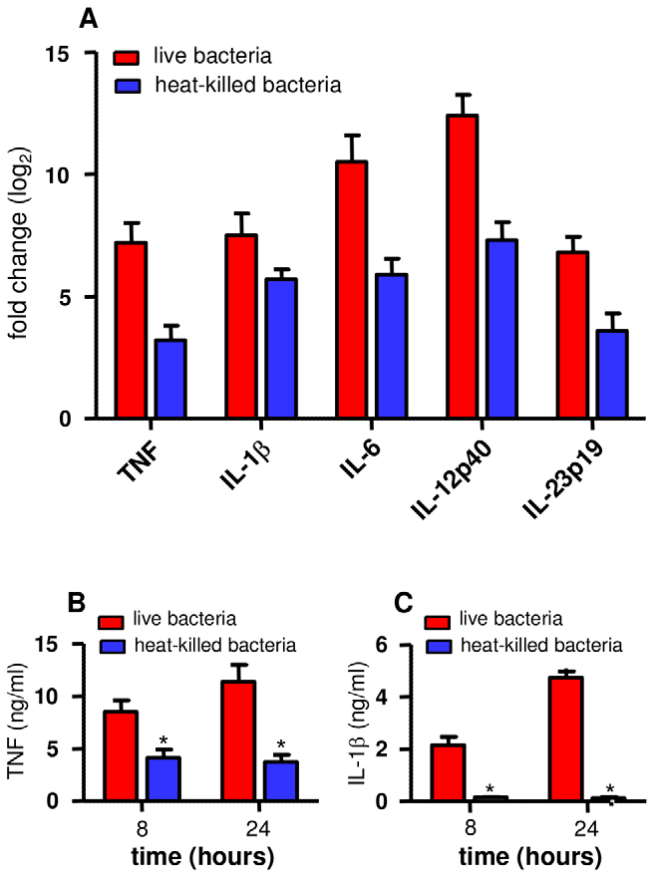
Bacterial viability and monocyte responses. Monocytes were stimulated with live or heat-killed *O. tsutsugamushi* for 8 (A) or 24 (B, C) hours. A. RNA was extracted, and qRT-PCR was performed to detect several genes that were differentially expressed in the microarray experiments. The results, expressed as the log_2_ ratio of fold changes, are presented as the mean ± SEM of two experiments performed in triplicate. B and C. Culture supernatants were analyzed for the presence of TNF (B) and IL-1β (C) using ELISAs. The results are expressed in ng/ml and are presented as the mean ± SD of two experiments performed in duplicate. *p<0.05.

We next focused on the genes involved in the immune response (Table S3). Approximately 15% of the genes that were upregulated in response to *O. tsutsugamushi* were cytokines (14 genes) or chemokines (14 genes). The expression of the genes encoding pro-inflammatory cytokines, such as TNF, IL-1β, IL-6, IL-12p35, IL-12p40, IL-23p19 and IL-15, and chemokines, such as CXCL10, CXCL11 and CCL20, was determined using qRT-PCR. After 8 hours of stimulation with *O. tsutsugamushi*, the expression of these genes was markedly increased (Figure 3A). This upregulation was sustained, as the expression was similar at 8 and 24 hours (Figure 3B). Next, we examined if the inflammatory response was dependent on bacterial viability. Monocytes were stimulated with live or heat-killed *O. tsutsugamushi* for 8 hours. In contrast to the genes involved in the type I IFN response, the expression of genes encoding pro-inflammatory cytokines, such as TNF, IL-1β, IL-6, IL-12p40 and IL-23p19, was upregulated in response to both live and heat-killed *O. tsutsugamushi*, although the expression level of these cytokines was partially reduced in monocytes incubated with heat-killed bacteria (Figure 4A). The transcriptional response induced in monocytes by *O. tsutsugamushi* was accompanied by cytokine production: *O. tsutsugamushi* stimulated high levels of TNF (Figure 4B) and IL-1β (Figure 4C) production by monocytes in a manner that correlated with gene expression. Heat-killed organisms also induced the production of TNF, although it was significantly (*p*<0.05) less than the production induced by live organisms (Figure 4B). In contrast, heat-killed organisms were unable to induce IL-1β production (Figure 4C), despite the increased IL-1β mRNA expression (see Figure 4A).

Other genes, including CD40, CD70 and CD80, which play a major role in macrophage-T cell interactions, and indoleamine-pyrrole 2,3 dioxygenase (INDO), a multi-functional protein that plays a role in the intracellular killing of bacteria, were upregulated in response to *O. tsutsugamushi* (Table S3). The upregulation of the gene encoding INDO was confirmed in monocytes from three donors using RT-PCR (Figure 3). Notably, the expression of several molecules, including CD14, CD22, CCR2, IL16, CLEC4A, CLEC10A and hepcidin antimicrobial peptides, was downregulated.

As the expression of a large number of chemokine and inflammatory cytokine genes associated with the M1 or M2 phenotype of macrophages was affected, we compared the transcriptomic profile of *O. tsutsugamushi*-infected monocytes with the list of M1 and M2 genes previously published [30]. We selected 32 and 28 genes representative of the M1 and M2 profiles, respectively (Table S4), in the genes associated with membrane receptors, cytokines, chemokines and apoptosis. Almost all of the genes characteristic of the M1 phenotype (29 of 32) were upregulated in response to *O. tsutsugamushi*. In contrast, M2 genes (except for CCL1, CCL23 and IL1RN) were either downregulated or remained unchanged. Taken together, these results demonstrate that *O. tsutsugamushi* induced a pro-inflammatory, M1 program in monocytes that did not require the presence of live organisms, which was in contrast to the type I IFN transcriptional signature.

### Specificity of the M1 program induced by *O. tsutsugamushi*

**Figure 5:**
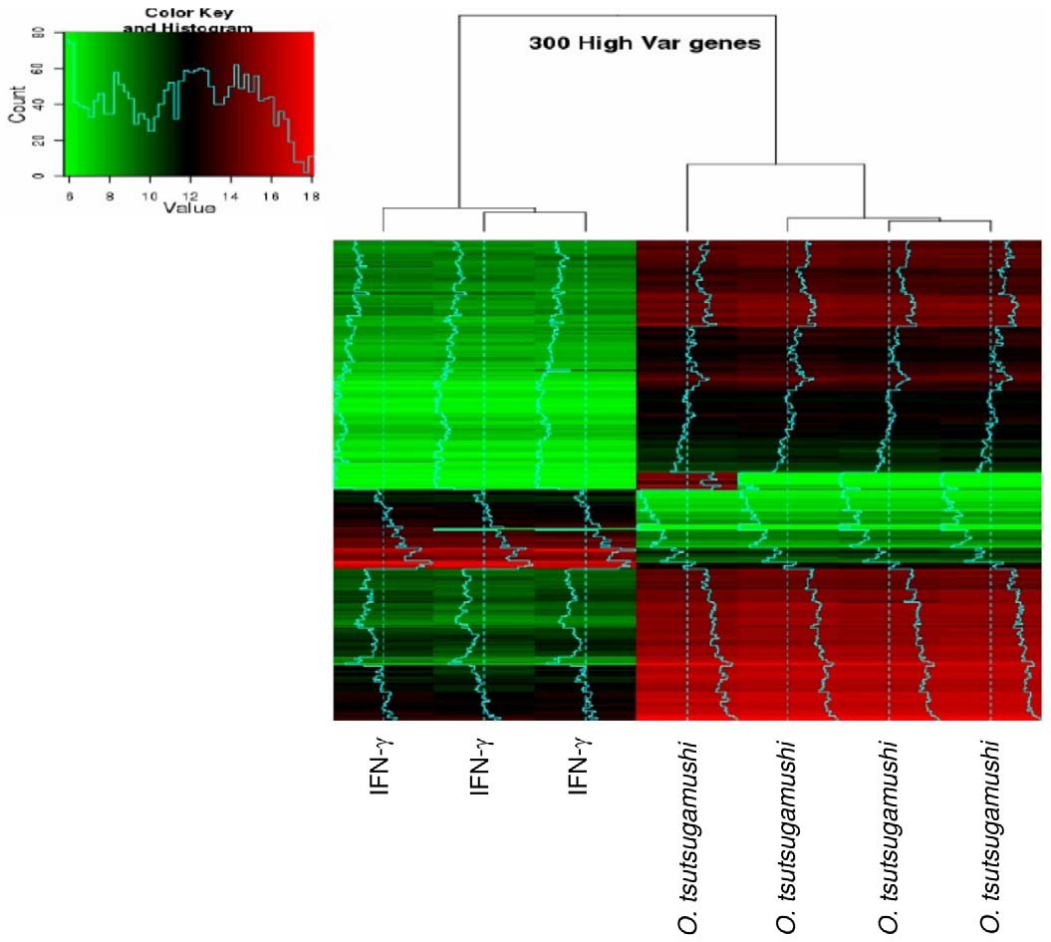
Hierarchical clustering of differentially expressed genes in stimulated monocytes. Monocytes were stimulated with *O. tsutsugamushi* or IFN-γ (500 UI/ml) for 8 hours, and genome-wide expression studies were performed using microarrays from Agilent Technologies. A hierarchical clustering consisting of 300 highly altered genes is shown.

As *O. tsutsugamushi* seems to induce an M1 program in monocytes, we compared this profile with the profile induced by IFN-γ, a canonical inducer of the M1 phenotype. The principal component analysis (Figure S2) and hierarchical clustering (Figure 5) revealed that the transcriptional pattern stimulated by *O. tsutsugamushi* was not identical to the program induced by IFN-γ. Approximately 76% of the genes altered in response to IFN-γ were also altered in response to *O. tsutsugamushi*, and 83% of the genes altered in response to *O. tsutsugamushi* were also altered in response to IFN-γ. Nevertheless, the expression of 572 and 413 genes was up- and downregulated in *O. tsutsugamushi*-stimulated cells compared with IFN-γ-stimulated cells, respectively. Among the genes upregulated in *O. tsutsugamushi*-stimulated monocytes, the GO analysis showed that genes involved in chromatin assembly, locomotion and lipid metabolism were enriched. Among the downregulated genes, we found a specific enrichment for genes in GO categories associated with cell activation, immune system processes and cell death. As determined using KEGG analysis, there was an over-representation of pathways associated with the immune response (14 of 18). Taken together, these findings suggest that while the response of monocytes to *O. tsutsugamushi* was polarized to an M1-like profile, it exhibited some specific features.

### *O. tsutsugamushi*-induced monocyte apoptosis

The analysis of genes upregulated in response to *O. tsutsugamushi* revealed the enrichment of genes in four GO categories related to apoptosis, including apoptosis, anti-apoptosis, the induction of apoptosis and the regulation of apoptosis (Table S5). In each GO category, several genes encoding TNF family members and regulators of apoptosis, such as caspases and Bcl-2, were upregulated. The ability of *O. tsutsugamushi* to induce the apoptosis of monocytes was studied using TUNEL staining. Although no apoptosis was detected in monocytes incubated for 6 hours with *O. tsutsugamushi*, after 24 h, 4±1% of monocytes were apoptotic, and this percentage increased to 8±1% after 48 h. In contrast, less than 1% of monocytes were apoptotic in the absence of *O. tsutsugamushi*. Interestingly, heat-killed *O. tsutsugamushi* did not induce monocyte apoptosis during the same incubation periods (Table 2). Taken together, our data show that *O. tsutsugamushi* induced an apoptosis-related gene program and the apoptosis of a minority of monocytes.

**Table 2:** *O. tsutsugamushi*-induced apoptosis.

Time (hours)	control	*O. tsutsugamushi*	heat-killed *O. tsutsugamushi*
6	not detectable	not detectable	not detectable
24	<1%	4±1%	<1%
48	<1%	8±1%	<1%

Monocytes were incubated with *O. tsutsugamushi* for different periods. Apoptosis was revealed by TUNEL assay and fluorescence microscopy. The results expressed as the ratio of TUNEL-positive cells and DAPI-stained nuclei ×100 are the mean ± SD of three different experiments.

### Host response genes in scrub typhus infection

**Figure 6:**
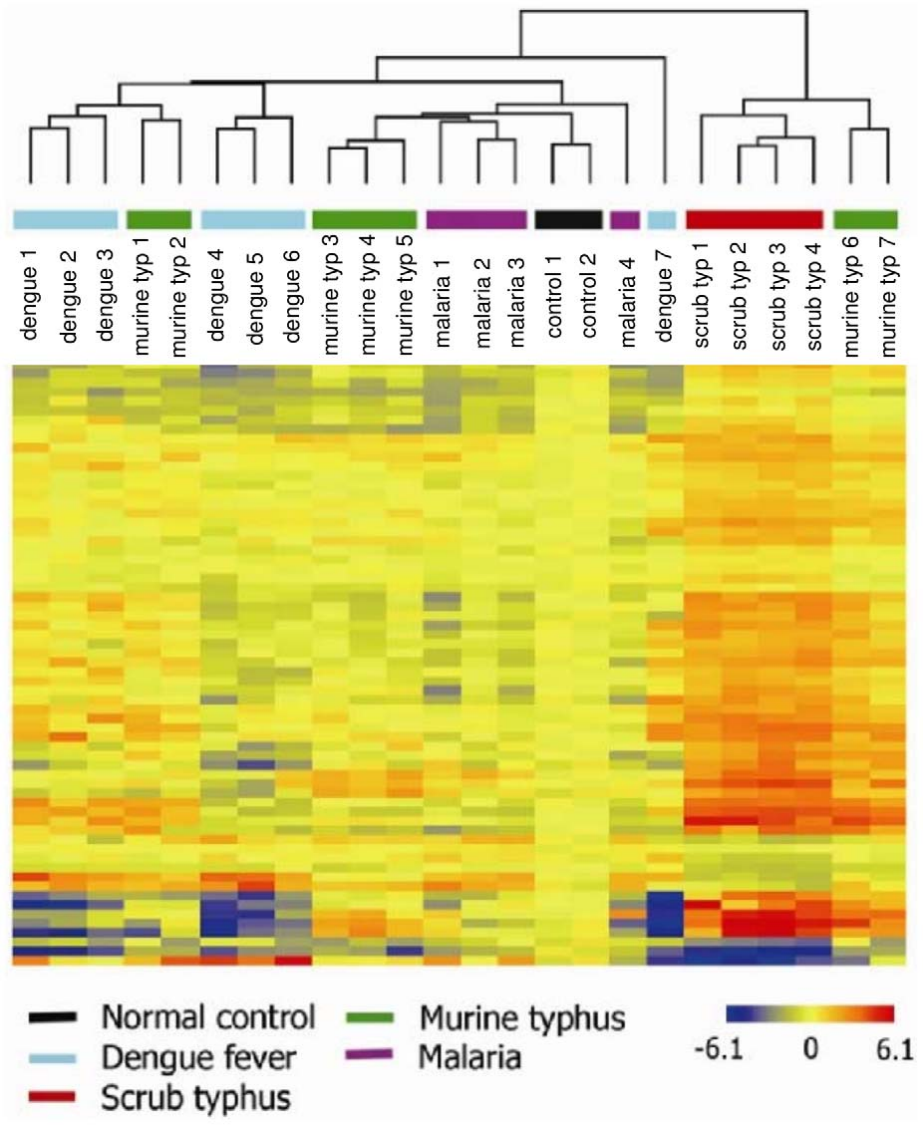
Hierarchical clustering in patients with scrub typhus. RNA was isolated from PBMCs from healthy controls and patients with different infectious diseases, and microarray studies were performed using Illumina Human-6 v2 BeadChips. The unsupervised hierarchical clustering of 22 patients and 2 RNA pools from healthy controls was performed based on the expression of 65 genes specific to scrub typhus (typ). The normalized expression level in each sample was baseline-adjusted to the mean expression level of the healthy control group and color-scaled, with red indicating increased expression and blue indicating decreased expression.

**Figure 7:**
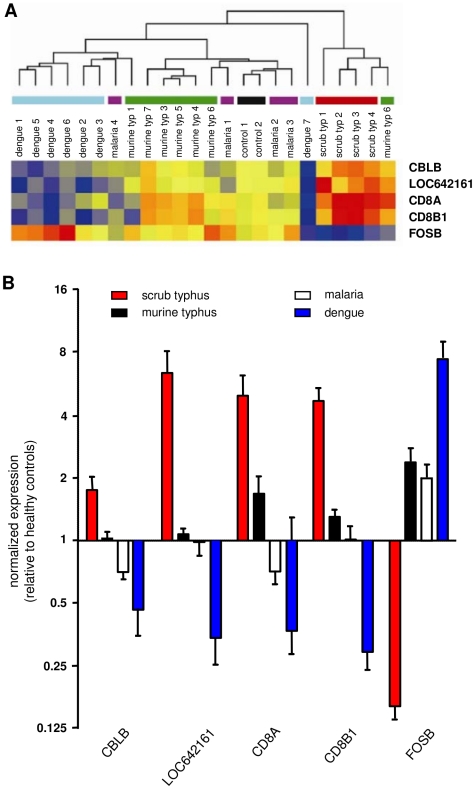
Reduced transcriptional signature of scrub typhus. The microarrays performed on RNA isolated from healthy controls or patients with infectious diseases suggested that five genes can be used as a specific signature of scrub typhus. A. The unsupervised hierarchical clustering of 22 patients and 2 pools of RNA from healthy controls was performed based on the expression of these five genes. The normalized expression level in each sample was baseline-adjusted to the mean expression level of the healthy control group and color-scaled, with red indicating increased expression and blue indicating decreased expression. B. The expression of the five genes was assessed using qRT-PCR. The results, expressed as the log_2_ ratio of fold changes, are presented as the mean ± SEM of all the patients in each group performed in duplicate.

The transcriptional pattern of PBMCs from patients with scrub typhus (n = 4) was compared to the pattern in pooled PBMCs isolated from healthy controls using microarrays. Using an absolute value of a FC greater than 2.0, we identified 613 and 517 transcripts that were up- and downregulated in scrub typhus patients, respectively. Most of the highly expressed genes corresponded to biological process categories including DNA metabolism, the cell cycle and cellular component organization and biogenesis (Table 3). Of particular interest, the upregulated genes involved in immune system process included IFN-γ (IFNG) and its related genes that encode absent in melanoma 2 (AIM2), guanylate binding protein 1(GBP1), IFN-γ-inducible protein 16 (IFI16) and indoleamine-pyrrole 2 (INDO). Significant enrichment in the downregulated genes was mainly observed in genes associated with immune-related processes, the inflammatory response and chemotaxis. To confirm the microarray-derived results, the level of 12 transcripts that were highly altered in patients with scrub typhus was re-assessed using qRT-PCR. The expression profiles detected using either technique were comparable, except for one gene, SSBP1. The transcriptomic profile of patients with scrub typhus was then compared to the profile of patients with murine typhus, malaria or dengue. Sixty-five probes corresponding to 63 genes were specifically expressed in patients with scrub typhus (*p*<0.05) (Table S6). The analysis of the microarray data by hierarchical clustering clearly showed that the four patients with scrub typhus were grouped together, separate from the other patients, while the transcriptional response of patients with murine typhus, malaria or dengue was more dispersed (Figure 6). These results clearly demonstrate that scrub typhus was characterized by a specific transcriptional signature. To reduce this transcriptional signature, the CBLB, LOC642161, CD8A and CD8B1 genes were selected because their expression was at least twofold greater in scrub typhus compared to the expression observed in the other infectious diseases; the FOSB gene was also selected because it was the only downregulated gene with the same fold difference. When hierarchical clustering was performed based on the expression of these five genes, the patients with scrub typhus were still grouped together, even though a patient with murine typhus also grouped in this cluster (Figure 7A). The expression of these five genes was then quantified using qRT-PCR and, as expected, the expression of CBLB, LOC64216, CD8A and CD8B1 was highly upregulated in patients with scrub typhus, while the expression of FOSB5 was downregulated (Figure 7B). This transcriptional profile was specific for scrub typhus, because the expression of these genes was completely different in murine typhus, malaria and dengue (Figure 7B).

**Table 3:** Enriched biological processes in scrub typhus.

Biological process^a^	Examples of genes
**Up-regulated genes**	
Immune system process	AIM2+, C1QA, C1QB, C1QC, CD164, CD8B1, CEACAM8, CLEC6A, CST7, CTLA4, CTSC, DPP4, FASLG, FCGR1A, GBP1, BP3, GBP4, GBP5, GPR65, IFI16+, IFI27+, IFNG, IL1R2, IL2RG, IL32, INDO+, OAS1+, OAS2+, OASL+, PSMB8+, PTPRC, RGS1, SERPING1, SPON2, TNFSF7, TUBB, TUBB2C
Response to stress	ATRX, BRCA1, CCNA2, CHAF1B, CHEK1, DCLRE1A, EXO1, FEN1, GTSE1, H2AFX, HMGB1, HMGB2, HSPA4, HSPB1, HSPD1, HSPE1, NEIL3, PCNA, POLE2, POLQ, PTTG1, RAD51, RAD51AP1, RAD54L, RECQL, SFPQ, TOP2A, TYMS, UHRF1
DNA metabolic process	CDC45L, CDC7, CDT1, DTYMK, KPNA2, MCM2, MCM4, MCM7, ORC1L, Pfs2,PRIM2A, RFC3, RNASEH2A, RRM2, TK1, TOP1
Cell cycle process	ASPM, BCAT1, BUB1, BUB1B, CCNB1, CCNB2, CCNF, CDC2, CDC20, CDC25A, CDC1, CDCA5, CDKN3, CENPE, CENPF, CET, CNAP1, E2F1, ESPL1, HCAP-G, KIF11, KIF15, KIF23, KIF2C, KNTC2, MACF1, MAD2L1, NEK2, PBK, PLK1, PTTG1, SMC2L1, SPAG5, STK6, STMN1, TPX2, TTK, TUBG1, UBE2C
Cellular component organization and biogenesis	BUB1B, CENPA, CKS2, GTSE1, GZMB, HIST1H1C, HIST1H2BD, HIST1H3C, KIF14, KIF20A, KIF4A, PACSIN1, PPP2R1B, RNF19, SMARCA5, TAF9, TUBA3, TUBB3, TUBB4Q, TUBB8, ZWINT
**Down-regulated genes**	
Immune system process	BCL2, BRDG1, CCL3L3, CD163, CD1C, CD79B, CLC, CLEC4A, CLEC4C, CSF1R, CXCL16, EBI2, FCER1A, FTH1, HLA-DMB, HLA-DOA, HLA-DOB, HLA-DRB1, HLA-DRB5, IL23A, IL4R, KLRB1, KLRF1, LILRA3, LTB, LY86, MS4A1, MS4A2, NR4A2, OSM, POU2F2, TCF7, TNF*, TNFRSF25, VIPR1
Defense response and inflammatory process	CD40LG, CD79A, CD83, CIAS1, EPHX2, FOS, IL1B, IL1RN, LY86, NALP1, NCR3, PLA2G7, PTX3, RNASE6, TCEA3, TLR10
Chemotaxis	CCL20, CCL3, CCL3L1, CCL4, CCL8, CCR3, CCR6, CCR7, CXCL1, CXCL16, CXCL2, IL1A, IL8, ROBO3

Enriched biological processes in scrub typhus with the list of modulated genes (FC>2.0) are shown.

a Determined by hypergeometric formula with Benjamini-Yekutieli correction [25], [26].

**+:** Genes induced by interferons.

### Relationship between the transcriptomic profiles detected in patients with scrub typhus and the profiles in *in vitro*-infected monocytes

**Figure 8:**
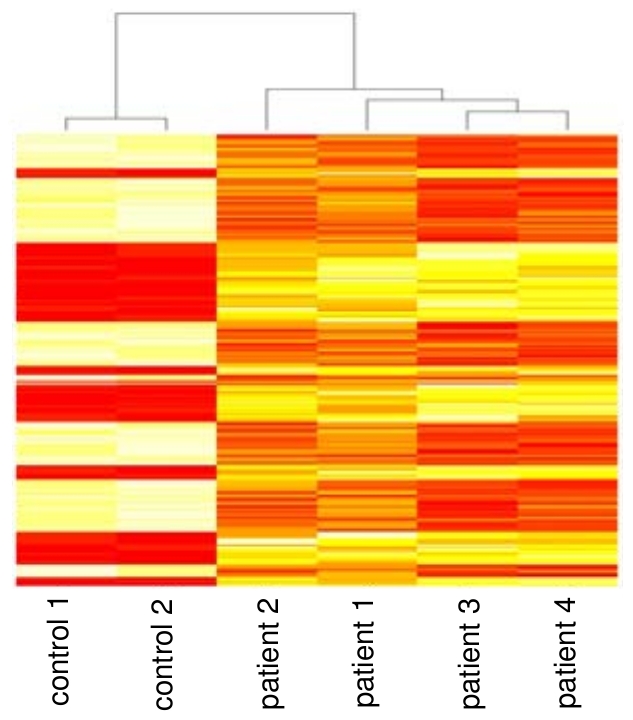
Comparison between patient blood samples and in vitro-infected monocytes. Monocytes from healthy donors were stimulated with *O. tsutsugamushi* for 8 hours, and Agilent microarrays were used to detect the differential expression of 2,015 genes that corresponded to 1,606 genes in the Illumina microarrays. Among these genes, 184 (250 probes) were altered in patients with scrub typhus with a *p* value less than 0.01. The hierarchical clustering of these genes demonstrates that the resulting transcriptional signature was specific to scrub typhus.

The transcriptional programs of PBMCs from patients with scrub typhus and those induced by *O. tutsugamushi* in monocytes were compared using Gene Symbol to allow the comparison between Illumina and Agilent data. The 2,015 probes differentially expressed in *O. tsutsugamushi*-stimulated monocytes detected using Agilent microarrays corresponded to 1,606 probes when Illumina assays were used. The differences were due to the fact that several Illumina probes are not annotated, leading to the impossibility to find these probes in Agilent probeset. In addition, Agilent probes were longer than those of Illumina. Interestingly, among the 1,606 probes that were modulated in *O. tsutsugamushi*-stimulated monocytes, 492 (*p*<0.05) and 184 probes (*p*<0.01) were also up- and downregulated, respectively, in patients with scrub typhus. This signature was clearly distinct from that of healthy controls (Figure 8). Among the 184 genes that were altered in scrub typhus, we found genes that were associated with important features of the monocyte response to *O. tsutsugamushi*, such as type I and II IFN and M1-associated genes (Table 3). These results suggest that the differential expression of genes in scrub typhus was related to *O. tsutsugamushi* infection.

## Discussion

Our study is the first report demonstrating the replication of *O. tsutsugamushi* in primary human monocytes; however, the efficiency of bacterial replication was lower than the replication observed in permissive cell lines, such as L929 cells. The sustained presence of bacteria within monocytes may be beneficial for the dissemination to target tissues, as *O. tsutsugamushi* is known for its high level of genetic and antigenic variability [2].

The interaction of *O. tsutsugamushi* with monocytes resulted in a transcriptomic pattern in which the expression of a large number of genes was altered. The first feature of the response was the polarization of monocytes towards an M1 phenotype. The M1 phenotype has been largely described in macrophages stimulated by IFN-γ, TNF and/or microbial products and has been associated with microbicidal competence and the skewing of the adaptive immune response towards Th1/Th17 responses [31], [32]. M1 phenotypes have been also described for macrophages stimulated with different bacterial pathogens including *Mycobacterium bovis* [33], *Legionella pneumophila* [34] and *Helicobacter pylori* [35] whereas *Mycobacterium tuberculosis* [36], *Coxiella burnetii* [37] and *Tropheryma whipplei* [29], [38] induce M2 profiles. We demonstrate that the transcriptomic profile of monocytes infected with *O. tsutsugamushi* was not identical to the profile observed following induction with IFN-γ because many genes were differentially expressed. The M1 polarization of monocytes infected with *O. tsutsugamushi* was persistent as demonstrated by the sustained upregulation of inflammatory genes. It may be related to the increased level of pro-inflammatory cytokines that has been detected in patients with scrub typhus [17], [18], [39], [40]. As activated or inflammatory monocytes play a major role in the dissemination of some pathogens, such as in the murine model of listeriosis [41] or human cytomegalovirus infection [42], [43], we hypothesize that a similar phenomenon may occur in scrub typhus. In addition to the induction of an M1 phenotype, *O. tsutsugamushi* upregulated the expression of genes involved in polarized immune responses. They included the genes encoding IL-6, IL-12p40, IL-23p19, GM-CSF and CCL20. As IL-6, IL-12p40, IL-23p19 and GM-CSF are important for Th17 proliferation and/or differentiation and that CCL20 binds to CCR6 selectively expressed on Th17 cells [44], we can hypothesize that *O. tsutsugamushi* orients the immune response to a Th17 phenotype. Note that IL-17 levels are higher in patients with scrub typhus than in healthy controls [45]. It is well known that Th1 responses are also critical to protection against intracellular pathogens [46]. It is likely that the uncontrolled Th1 and Th17 responses to *O. tsutsugamushi* contribute to the pathophysiology of scrub typhus [47], [48].

The second feature of the transcriptional program induced by *O. tsutsugamushi* in human monocytes was the upregulation of genes belonging to the “response to virus” GO category; these genes essentially corresponded to the type I IFN genes and ISGs. The release of IFN-β and the expression of ISGs have been reported in response to LPS and intracellular bacteria, such as *Chlamydia* sp., *Salmonella enteritica* serovar typhimurium [49], *T. whipplei* [50] and *Francisella tularensis* [51]. We recently reported that *Rickettsia prowazekii*, an intracellular bacterium related to *O. tsutsugamushi*, stimulates a type I IFN response in endothelial cell lines [52]. *Listeria monocytogenes* stimulates an IRF-3-dependent cytosolic response consisting of IFN-β and several ISGs [53]. It is tempting to speculate that the production of IFN-β and the expression of ISGs, at least in part, are related to the cytosolic location of the bacteria. Indeed, *L. monocytogenes* [54] and *O. tsutsugamushi* [55] are known to reside in the cytosol. Among the genes upregulated in response to *O. tsutsugamushi*, we detected TBK-1 and IRF-7. TBK-1 is involved in IFN-β production after cytoplasmic recognition of *L. monocytogenes* [56], and IRF-7 is increased after infection and promotes an amplification loop of IFN-β production [49]. It is likely that a substantial number of genes included in the “response to virus” GO category are controlled by IFN-β and not directly dependent on *O. tsutsugamushi* infection. In LPS-stimulated cells, a considerable number of the differentially expressed genes are due to type I IFN synthesis and signaling through IFN receptors [49]. The effects of type I IFNs can be beneficial or detrimental to host defense against bacterial infections [49]. Type I IFNs activate NK cells and cytotoxic T cells (CTLs), which are critical to clear cytosolic pathogens, including *Rickettsia* spp. [57], [58]. In addition, type I IFNs sensitize the host cells to apoptosis [59] through the induction of ISGs, such as TRAIL, FAS, XIAP-associated factor-1 (XAF-1), caspase-8, protein kinase R (PKR), 2′5′oligoadenylate synthase (OAS), phospholipid scramblase and the promyelocytic leukemia gene product [60]. All of these genes were upregulated in monocytes infected with *O. tsutsugamushi*. In contrast, type I IFNs are detrimental to the host during *L. monocytogenes* infection. They contribute to macrophage cell death [61] and sensitize T lymphocytes to apoptosis induced by listeriolysin O [62]. As a result, IRF3^−/−^ and IFNAR^−/−^ mice show increased resistance to *L. monocytogenes* compared to wild type mice [56]. IFN-β has been shown to inhibit the in vitro replication of *Francisella tularensis* in murine macrophages [63]. Recently, our group has shown that the type I IFN response is detrimental to murine macrophages infected with *Tropheryma whipplei*. Indeed, macrophage apoptosis and bacterial replication are inhibited in IFNAR^−/−^ macrophages compared with wild type macrophages [50]. A previous study has shown that type I IFN inhibited *O. tsutsugamushi* replication depending on the bacterial strain and the genetic background of host cells [64]. However, further studies are required to determine the exact role of type I IFNs in *O. tsutsugamushi* infection.

The third prominent feature of the infection of human monocytes with *O. tsutsugamushi* was the enrichment in apoptosis-related genes but only a minority of *O. tsutsugamushi*-infected monocytes were apoptotic. Cell death has already been described in lymphocytes, lymph nodes and endothelial cell lines in response to *O. tsutsugamushi* [65]. In vivo, cell death is prominent in mice susceptible to *O. tsutsugamushi*, but it is not detected in resistant mice [65]. Our results were apparently contradictory with those of Kim et al. obtained with the THP-1 macrophage cell line in which *O. tsutsugamushi* inhibits transiently the cell death induced by apoptosis promoters. In addition, the ability to prevent apoptosis is not related to bacterial virulence [66]. We hypothesized that the apparent discrepancy between gene expression and low level of apoptosis is associated to the modulation of the genes belonging to apoptosis and anti-apoptosis GO terms in inflammatory conditions [67]. The precise mechanisms used by *O. tsutsugamushi* to affect the cell death of monocytes remain to be determined. Apoptosis can result from inflammasome activation that involves caspase-1 activation and IL-1β secretion [68]. In this study, we demonstrate that live *O. tsutsugamushi* induced IL-1β secretion by monocytes, whereas heat-killed bacteria stimulated the expression of the gene encoding IL-1β but did not induce secretion of active IL-1β. Similar to what has been reported for *F. tularensis* [51] and *T. whipplei* [50], type I IFNs may promote apoptosis in monocytes infected with *O. tsutsugamushi*. We hypothesize that *O. tsutsugamushi* stimulates inflammasome activation via IFN-β release when the bacteria reach the cytosol.

Lastly, we extended our analysis of the host response to *O. tsutsugamushi* to patients with scrub typhus. For that purpose, we analyzed the transcriptional response of whole PBMCs from patients with scrub typhus and then compared this response to that observed in monocytes stimulated with *O. tsutsugamushi*. In patients with scrub typhus, we observed a significant upregulation of IFN-γ, a type II IFN, and its related genes, suggesting an important role for the type II IFN pathway in the response to *O. tsutsugamushi* infection. IFN-γ is a key cytokine in macrophage activation and Th1 responses, and these cells are necessary to clear *O. tsutsugamushi* infection [20]. IFN-γ can also directly exert an inhibitory effect on the intracellular replication of *O. tsutsugamushi* in non-immune cells [64]. Indeed, increased IFN-γ production correlates with the acquisition of resistance to *O. tsutsugamushi* infection in immune mice [69]. We hypothesize that the increased production of IFN-γ is, at least in part, a consequence of the elevated number of CD8^+^ T cells and NK cells observed in patients with scrub typhus [70]. This hypothesis is also in agreement with the increased expression of genes encoding the CD8 subunits and the abundance of gene transcripts involved in cell cycle and cell division. All together, these data emphasize the role of type II IFN and cell-mediated immunity in the protection against *O. tsutsugamushi* infection.

The transcriptional signature of patients with scrub typhus included the differential expression of the CBLB, LOC642161, CD8A, CD8B1 and FOSB genes, when compared to healthy controls and patients with other infectious diseases. Among these genes, CBLB is interesting, as there is increasing evidence that it has important functions in the immune system. The CBLB gene encodes the E3 ubiquitin ligase, Cbl-b, which controls peripheral T cell activation and tolerance by regulating CD28 co-stimulatory signaling [71], [72]. In infectious diseases, the increased expression of Cbl-b in many chronic infections is believed to be due to a defective immune response [15]–[17]. Cbl-b also modulates the stability of bacterial effector proteins essential for virulence, as recently reported in *Pseudomonas aeruginosa* infection [24]. The enhanced expression of Cbl-b in patients with scrub typhus may suggest a role for it in the degradation of bacterial products or in the immune evasion of *O. tsutsugamushi*, but it may also represent a regulatory mechanism of the immune response to prevent over-activation of T cells. Independent of the putative role of these five genes in the pathophysiology of scrub typhus, their specific alteration in scrub typhus may be useful to improve the diagnosis of this infection.

In conclusion, we report the global transcriptional response of monocytes in response to *O. tsutsugamushi*. *O. tsutsugamushi* induced a specific M1 phenotype and stimulated a type I IFN response. Type I and II IFNs and the M1 signature were also found in the PBMCs from patients with scrub typhus, suggesting that these molecules may be associated with the inflammatory complications of scrub typhus.

## Supporting Information

Figure S1 Time course of gene modulation.
(PDF)

Figure S2 PCA and Venn diagram.
(PDF)

Table S1 Characteristics of patients in each group of infectious diseases.
(PDF)

Table S2 Nucleotide sequences of oligonucleotide primers.
(PDF)

Table S3 Modulated genes in the “immune response” GO term.
(PDF)

Table S4 M1 and M2 genes in *O. tsutsugamushi*-stimulated monocytes.
(PDF)

Table S5 Apoptosis-related genes in *O. tsutsugamushi*-stimulated monocytes.
(PDF)

Table S6 List of the 65 transcripts specific for scrub typhus.
(PDF)
