# Correction: *Orientia tsutsugamushi* Stimulates an Original Gene Expression Program in Monocytes: Relationship with Gene Expression in Patients with Scrub Typhus

**DOI:** 10.1371/journal.pntd.0014279

**Published:** 2026-05-04

**Authors:** The PLOS Neglected Tropical Diseases Editors

The *PLOS Neglected Tropical Diseases* Editors issue this notice to resolve the previously published Expression of Concern on this article [1,2] and update the article’s Materials and Methods section.

The Ethics Statement for [1] is updated to:

Blood samples from patients and controls were collected after informed and written consent obtained from each participant, and the study was conducted with the approval of the Human Research Ethics Committee of the Faculty of Medicine, Siriraj Hospital, Bangkok, Thailand (reference number 282/2007, issued on 24 August 2007).

In addition, the following sentence is added to the start of the Patients section:

The samples used in this study were collected between August 2007 and July 2008. All participants were 18 years or older.

This Correction supersedes the prior Expression of Concern [2].
