# Supplementary figures and images for: Orientia tsutsugamushi Stimulates an Original Gene Expression Program in Monocytes: Relationship with Gene Expression in Patients with Scrub Typhus
Source: PLoS Negl Trop Dis. 2011 May 17;5(5):e1028. doi: 10.1371/journal.pntd.0001028 (PMC3096591; doi:10.1371/journal.pntd.0001028)

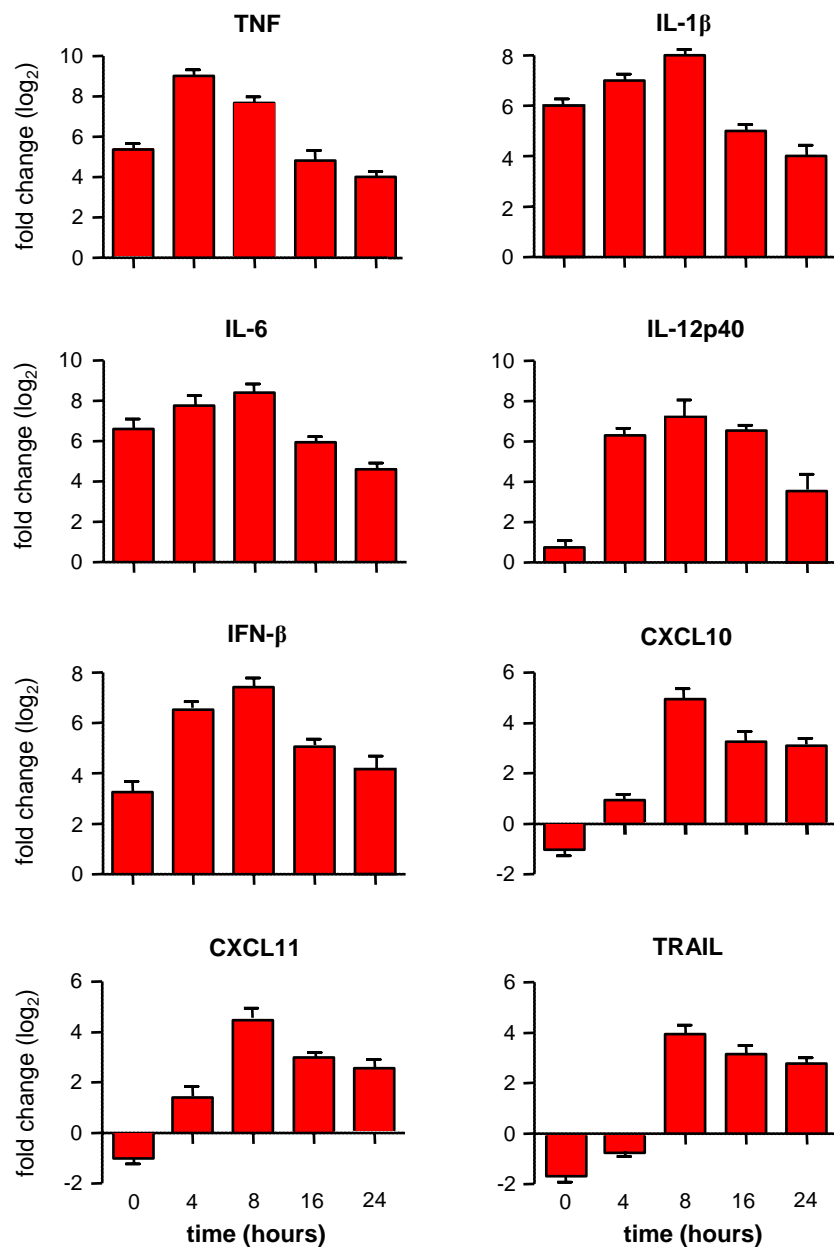

new Figure S1

Supplement: Figure S1 — Time course of gene modulation. (PDF) [file pntd.0001028.s001.pdf]

**Figure S2. PCA and Venn diagram**

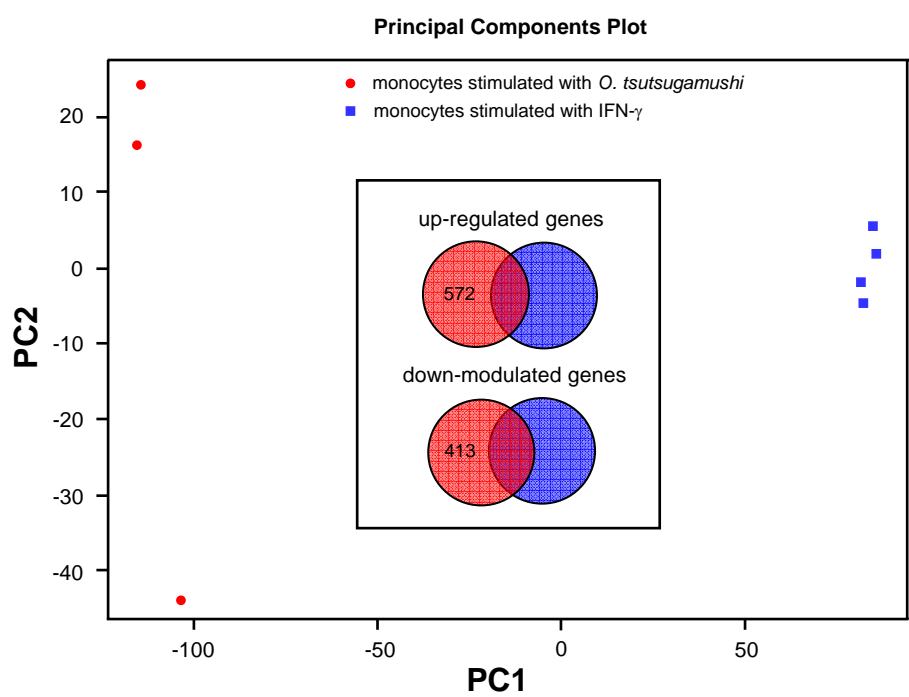

Supplement: Figure S2 — PCA and Venn diagram. (PDF) [file pntd.0001028.s002.pdf]
