# Supplementary material for: Orientia tsutsugamushi Stimulates an Original Gene Expression Program in Monocytes: Relationship with Gene Expression in Patients with Scrub Typhus
Source: PLoS Negl Trop Dis. 2011 May 17;5(5):e1028. doi: 10.1371/journal.pntd.0001028 (PMC3096591; doi:10.1371/journal.pntd.0001028)
Supplement: Table S1 — Characteristics of patients in each group of infectious diseases. (PDF) [file pntd.0001028.s003.pdf]

Table S1. Characteristics of patients in each group of infectious diseases

	scrub typhus	murine typhus	malaria	dengue
Number	4	7	4	7
Age	46 ± 18.4	41.7 ± 14.5	26.5 ± 6.0	31.9 ± 18.1
Male : Female	2:2	1:6	2:2	4:3
Mean fever day (range)	10.5 (3-20)	9.7 3-15	5.3 (3-7)	2.3 (1-4)*
Body temperature (°C)	38.0 ± 0.8	37.8 ± 1.0	38.8 ± 0.6	38.9 ± 0.9
WBC (x 10^3/μl)	13.5 ± 2.4	8.1 ± 2.4**	4.1 ± 1.3***	5.1 ± 2.6***
Platelets (x 10^3/μl)	182.5 ± 69.3	221.7 ± 120.1	37 ± 19.1	131.33 ± 65.34
Neutrophils (%)	76.8 ± 9.7	72.1 ± 12.4	67.6 ± 9.0	65.2 ± 20.6
Lymphocytes (%)	17.4 ± 12.1	20.8 ± 12.1	17.9 ± 11.4	16.8 ± 15.7
Monocytes (%)	4.3 ± 2.5	5.1 ± 2.1	7.1 ± 2.5	6.9 ± 4.5
Lymphocytes/Monocytes	2.0 ± 4.9	4.1 ± 5.8	2.5 ± 4.6	2.4 ± 3.8

Data are mean values of each group of patients with standard deviation.

*P < 0.05, **P < 0.01, ***P < 0.001, as compared to the scrub typhus group.
