# Supplementary material for: Orientia tsutsugamushi Stimulates an Original Gene Expression Program in Monocytes: Relationship with Gene Expression in Patients with Scrub Typhus
Source: PLoS Negl Trop Dis. 2011 May 17;5(5):e1028. doi: 10.1371/journal.pntd.0001028 (PMC3096591; doi:10.1371/journal.pntd.0001028)
Supplement: Table S2 — Nucleotide sequences of oligonucleotide primers. (PDF) [file pntd.0001028.s004.pdf]

Table S2. Nucleotide sequences of oligonucleotide primers

Symbol	Forward primers	Reverse primers
TNF	5'- CCCGACTATCTCGACTTTGC-3'	5'-AGGTTGAGGGTGTCTGAAGGA-3'
IL1β	5'-GCTGAGGAAGATGCTGGTTC-3'	5'-TCCATATCCTGTCCCTGGAG-3'
IL6	5'-TACCCCCAGGAGAAGATTCC-3'	5'-TTTTCTGCCAGTGCCTCTTT-3'
IL12p35	5'-TCAGCAACATGCTCCAGAAGGC-3'	5'-TGCATTCATGGTCTTGAACTCCACC-3'
IL12p40	5'-AGGGGACAACAAGGAGTATGAGT-3'	5'-AGGGAGAAGTAGGAATGTGGAGT-3'
IL23p19	5'-AGGAGAAGAGGGAGATGAAGAGAC-3'	5'-GCTATCAGGGAGCAGAGAAGG-3'
IL15	5'-GTTAGCAGATAGCCAGCCCATAC-3'	5'-TACTCAAAGCCACGGTAAATCC-3'
CXCL10	5'-AAGCAGTTAGCAAGGAAAGGTC-3'	5'-TTGAAGCAGGGTCAGAACATC-3'
CXCL11	5'-TATAGCCTTGGCTGTGATATTGTG-3'	5'-CTGCCACTTTCACTGCTTTTACC-3'
CCL20	5'-GACATCAATGCTATCATCTTTCAC-3'	5'-GCTATGTCCAATTCCATTCCA-3'
IFNβ	5'-GACGCCGCATTGACCATCTA-3'	5'-CCTTAGGATTTCCACTCTGACT-3'
IFNα8	5'-ATGACCTGGAGTCCTGTGTGAT-3'	5'-GATCTCATGATTTCTGCTCTGACAAC-3'
OAS1	5'-TCCGCCTAGTCAAGCACTGGTA-3'	5'-CCTGGGCTGTGTTGAAATGTGT-3'
MX1	5'-GCCACCACAGAGGCTCTCAG-3'	5'-CTCAGCTGGTCCTGGATCTCCT-3'
INDO	5'-TGCTGGTGGAGGACATGCTG-3'	5'-TGAAAGGACAAACTCACGGACTGA-3'
TLR5	5'-CTTGTCCCAGTACCAGTTGATGA-3'	5'-AGGAGATGGTTGCTACAGTTTG-3'
CBLB	5'-CCCTTTGTTGATCTAGCAAGTG-3'	5'-GTGCCTGTGAACCATCTGAA-3'
LOC642161	5'-CTCAAAATGCCCCTCCTT TC-3'	5'-GGTTCTGTGAGTCCTGCTT-3'
CD8A	5'-CCTTTACTGCAACCACAGGA-3'	5'-AGGAAGGATCTCAGTTTGAAG-3'
CD8B1	5'-ACTTCTGCATGATCGTCG G-3'	5'-AGGGTGGACTTCTTGGTG-3'
FOSB	5'-CAGCAGCTAAATGCAGGA-3'	5'-TTTGGAGCTCGGCGATCT-3'
