# Supplementary material for: Orientia tsutsugamushi Stimulates an Original Gene Expression Program in Monocytes: Relationship with Gene Expression in Patients with Scrub Typhus
Source: PLoS Negl Trop Dis. 2011 May 17;5(5):e1028. doi: 10.1371/journal.pntd.0001028 (PMC3096591; doi:10.1371/journal.pntd.0001028)
Supplement: Table S3 — Modulated genes in the “immune response” GO term. (PDF) [file pntd.0001028.s005.pdf]

Table S3. Modulated genes in the "immune response" GO term

Gene function and full gene name	Gene symbol	GenBank ID	FC
adenosine deaminase	ADA	NM_000022	3.8
absent in melanoma 2	AIM2	NM_004833	8.1
aquaporin 9	AQP9	AQP9	3.6
**chemokine (C-C motif) ligand 1**	**CCL1**	**NM_002981**	**20.3**
**chemokine (C-C motif) ligand 3**	**CCL3**	**NM_002983**	**9.0**
**chemokine (C-C motif) ligand 4**	**CCL4**	**NM_002984**	**16.9**
**chemokine (C-C motif) ligand 7**	**CCL7**	**NM_006273**	**23.2**
**chemokine (C-C motif) ligand 8**	**CCL8**	**NM_005623**	**74.9**
**chemokine (C-C motif) ligand 20**	**CCL20**	**NM_004591**	**13.6**
CD274 molecule	CD274	NM_014143	16.6
CD40 molecule, TNF receptor superfamily member 5	CD40	NM_001250	6.0
CD70 molecule	CD70	NM_001252	3.8
CD80 molecule	CD80	NM_005191	21.3
C-type lectin domain family 4, member D	CLEC4D	NM_080387	5.6
C-type lectin domain family 4, member E	CLEC4E	NM_014358	7.5
colony stimulating factor 2 (granulocyte-macrophage)	CSF2	NM_000758	7.8
colony stimulating factor 3 (granulocyte)	CSF3	NM_000759	97.5
**chemokine (C-X-C motif) ligand 1 (melanoma growth stimulating activity, alpha)**	**CXCL1**	**NM_001511**	**4.8**
**chemokine (C-X-C motif) ligand 2**	**CXCL2**	**NM_002089**	**2.8**
**chemokine (C-X-C motif) ligand 3**	**CXCL3**	**NM_002090**	**5.6**
**chemokine (C-X-C motif) ligand 5**	**CXCL5**	**NM_002994**	**2.7**
**chemokine (C-X-C motif) ligand 10**	**CXCL10**	**NM_001565**	**16.3**
**chemokine (C-X-C motif) ligand 11**	**CXCL11**	**NM_005409**	**98.2**
**chemokine (C-X-C motif) ligand 13 (B-cell chemoattractant)**	**CXCL13**	**NM_006419**	**2.3**
**chemokine (C-X-C motif) ligand 14**	**CXCL14**	**NM_004887**	**4.1**
eukaryotic translation initiation factor 2-alpha kinase 2	EIF2AK2	NM_002759	13.0
Fas (TNF receptor superfamily, member 6)	FAS	NM_000043	3.3
guanylate binding protein 1, interferon-inducible, 67kDa	GBP1	NM_002053	5.9
guanylate binding protein 3	GBP3	NM_018284	5.2
guanylate binding protein 5	GBP5	NM_052942	4.5
guanylate binding protein 7	GBP7	NM_207398	15.4
GTP binding protein overexpressed in skeletal muscle	GEM	NM_005261	3.0
GTP binding protein 1	GTPBP1	NM_004286	3.8
interferon, alpha-inducible protein 27	IFI27	NM_005532	24.1
interferon-induced protein 35	IFI35	NM_005533	7.6
interferon, alpha-inducible protein 6	IFI6	NM_022873	10.2
interferon-induced protein with tetratricopeptide repeats 1	IFIT1	NM_001548	100.0
interferon-induced protein with tetratricopeptide repeats 2	IFIT2	NM_001547	49.2
interferon-induced protein with tetratricopeptide repeats 3	IFIT3	NM_001549	31.3
interferon-induced protein with tetratricopeptide repeats 5	IFIT5	NM_012420	9.7
interferon induced transmembrane protein 1 (9-27)	IFITM1	NM_003641	14.7
interferon induced transmembrane protein 2 (1-8D)	IFITM2	NM_006435	8.8
interferon induced transmembrane protein 3 (1-8U)	IFITM3	NM_021034	10.6
**interleukin 12B (p40)**	**IL 12B**	**NM_002187**	**> 100**
**interleukin 15**	**IL15**	**NM_172174**	**3.6**
**interleukin 18 (interferon-gamma-inducing factor)**	**IL18**	**NM_001562**	**3.7**
**interleukin 19**	**IL19**	**NM_153758**	**35.9**

**interleukin 1, alpha**	**IL1A**	**NM_000575**	**9.0**
**interleukin 1, beta**	**IL1B**	**NM_000576**	**20.7**
**interleukin 1 family, member 5 (delta)**	**IL1F5**	**NM_012275**	**28.3**
**interleukin 1 family, member 9**	**IL1F9**	**NM_019618**	**100.0**
**interleukin 1 receptor antagonist**	**IL1RN**	**BC068441**	**17.8**
**interleukin 1 receptor antagonist**	**IL1RN**	**NM_173842**	**37.9**
**interleukin 7 receptor**	**IL7R**	**NM_002185**	**4.5**
**interleukin 6**	**IL6**	**NM_000600**	**> 100**
**interleukin 8**	**IL8**	**NM_000584**	**2.7**
**interleukin 23, alpha subunit p19**	**IL23A**	**NM_016584**	**5.1**
indoleamine-pyrrole 2,3 dioxygenase	INDO	NM_002164	15.6
interferon regulatory factor 2	IRF2	NM_002199	2.6
interferon regulatory factor 7	IRF7	NM_004031	14.9
ISG15 ubiquitin-like modifier	ISG15	NM_005101	54.1
MHC class I polypeptide-related sequence B	MICB	NM_005931	2.9
myxovirus (influenza virus) resistance 1, interferon-inducible protein p78 (mouse)	MX1	NM_002462	26.7
myxovirus (influenza virus) resistance 2 (mouse)	MX2	NM_002463	50.2
2',5'-oligoadenylate synthetase 1, 40/46kDa	OAS1	NM_002534	13.6
2'-5'-oligoadenylate synthetase 2, 69/71kDa	OAS2	NM_016817	26.2
2'-5'-oligoadenylate synthetase 3, 100kDa	OAS3	NM_006187	21.7
2'-5'-oligoadenylate synthetase-like	OASL	NM_003733	36.3
oncostatin M	OSM	NM_020530	4.2
regulator of G-protein signaling 1	RGS1	NM_002922	4.7
sema domain, immunoglobulin domain (Ig), short basic domain, secreted, (semaphorin) 3C	SEMA3C	NM_006379	2.7
transporter 1, ATP-binding cassette, sub-family B (MDR/TAP)	TAP1	NM_000593	2.9
tumor necrosis factor receptor superfamily, member 4	TNFRSF4	NM_003327	4.8
tumor necrosis factor receptor superfamily, member 9	TNFRSF9	NM_001561	5.8
tumor necrosis factor (ligand) superfamily, member 10	TNFSF10	NM_003810	78.7
tumor necrosis factor (ligand) superfamily, member 15	TNFSF15	NM_005118	10.4
tumor necrosis factor (ligand) superfamily, member 9	TNFSF9	NM_003811	8.1
tripartite motif-containing 22	TRIM22	NM_006074	8.4
chemokine (C-C motif) receptor 2	CCR2	NM_000647	-2.5
chemokine (C-C motif) receptor 2	CCR2	NM_000648	-2.8
CD14 molecule	CD14	NM_000591	-2.9
CD22 molecule	CD22	NM_001771	-3.7
class II, major histocompatibility complex, transactivator	CIITA	U18259	-3.6
C-type lectin domain family 10, member A	CLEC10A	NM_182906	-4.4
C-type lectin domain family 4, member A	CLEC4A	NM_016184	-3.6
cytotoxic and regulatory T cell molecule	CRTAM	NM_019604	-2.7
hepcidin antimicrobial peptide	HAMP	NM_021175	-4.7
major histocompatibility complex, class II, DM beta	HLA-DMB	BC035650	-10.2
interleukin 16 (lymphocyte chemoattractant factor)	IL16	NM_172217	-5.4
interleukin 16 (lymphocyte chemoattractant factor)	IL16	NM_004513	-3.3
Notch homolog 1, translocation-associated (Drosophila)	NOTCH1	NM_017617	-4.0
ring finger protein 125	RNF125	NM_017831	-5.4
signaling threshold regulating transmembrane adaptor 1	SIT1	NM_014450	-4.1
transcription factor 7 (T-cell specific, HMG-box)	TCF7	NM_003202	-2.5
tumor necrosis factor (ligand) superfamily, member 11	TNFSF11	NM_003701	-2.1

The genes encoding chemokines and cytokines were presented in bold.
