# Supplementary material for: Orientia tsutsugamushi Stimulates an Original Gene Expression Program in Monocytes: Relationship with Gene Expression in Patients with Scrub Typhus
Source: PLoS Negl Trop Dis. 2011 May 17;5(5):e1028. doi: 10.1371/journal.pntd.0001028 (PMC3096591; doi:10.1371/journal.pntd.0001028)
Supplement: Table S4 — M1 and M2 genes in O. tsutsugamushi-stimulated monocytes. (PDF) [file pntd.0001028.s006.pdf]

Table S4. M1 and M2 genes in *O. tsutsugamushi*-stimulated monocytes

Gene category and name	Gene symbol	Fold change
**M1 genes**		
Chemokine (C-C motif) receptor 7	CCR7	2.6
Interleukin 2 receptor α chain	IL2RA	1.9
Interleukin 7 receptor	IL7R	4.5
Interleukin 15 receptor α chain	IL15RA	15.6
Chemokine (C-C motif) ligand 2	CCL2	nc
Chemokine (C-C motif) ligand 3	CCL3	9.0
Chemokine (C-C motif) ligand 4	CCL4	16.9
Chemokine (C-C motif) ligand 5	CCL5	1.5
Chemokine (C-C motif) ligand 15	CCL15	nc
Chemokine (C-C motif) ligand 19	CCL19	nc
Chemokine (C-C motif) ligand 20	CCL20	13.6
Chemokine (C-X-C motif) ligand 8	CXCL8, IL8	2.7
Chemokine (C-X-C motif) ligand 9	CXCL9	nc
Chemokine (C-X-C motif) ligand 10	CXCL10	16.3
Chemokine (C-X-C motif) ligand 11	CXCL11	98.1
Chemokine (C-X-C motif) ligand 16	CXCL16	nc
Endothelial cell growth factor 1 (platelet-derived)	ECGF1	1.6
Interleukin 1α	IL1A	9.0
**Interleukin 1β**	**IL1B**	**20.6**
**Interleukin 6**	**IL6**	**> 100**
**Interleukin 12A**	**IL12A**	**3.0**
**Interleukin 12B**	**IL12B**	**> 100**
**Interleukin 15**	**IL15**	**3.6**
**Interleukin 23 α subunit p19**	**IL23A**	**5.1**
**Tumor necrosis factor ligand superfamily, member 2**	**TNF**	**13.9**
Tumor necrosis factor ligand superfamily, member 10	TRAIL	78.7
Visfatin	PBEF1	7.0
**Apoptosis-related genes**		
BCL2-related protein A1	BCL2A1	nc
Fas (TNF receptor superfamily, member 6)	FAS	3.3
Baculoviral IAP repeat-containing 3	BIRC3	1.5
growth arrest and DNA-damage-inducible, gamma	GADD45G	2.0
XIAP associated factor-1	XAF1	13.2
**M2 genes**		
CD36	CD36	-2
CD163	CD163	-1.8
CD209	DCSIGN	nc
Chemokine (C-X-C motif) receptor 4	CXCR4	-3.8
C-type lectin receptor DCL-1	DCL-1	-1.5
C-type lectin superfamily member 12	DECTIN1	-1.9
Fcε-RII	CD23	-2.5
G protein-coupled receptor 105	P2RY14	-1.7
G protein-coupled receptor 86	GPR86	-5.1
Histamine receptor H1	HRH1	1.7
macrophage scavenger receptor 1	MSR1	1.7
Macrophage scavenger receptor 1	MSR1	nc
Mannose receptor C type 1	MRC1	-2.2
Membrane-spanning 4-domains, subfamily A, member 4	MS4A4A	-1.7
Membrane-spanning 4-domains, subfamily A, member 6A	MS4A6A	-3
Purinergic receptor P2Y5	P2RY5	-1.6
Transforming growth factor β receptor II	TGFBR2	-3.6

Transforming growth factor β receptor II	TGFBR2	-3.9
Chemokine (C-C motif) ligand 1	CCL1	20.3
Chemokine (C-C motif) ligand 13	CCL13	2.0
Chemokine (C-C motif) ligand 17	CCL17	nc
Chemokine (C-C motif) ligand 18	CCL18	nc
Chemokine (C-C motif) ligand 22	CCL22	nc
Chemokine (C-C motif) ligand 23	CCL23	4.7
Chemokine (C-C motif) ligand 24	CCL24	nc
Interleukin 1 receptor antagonist	IL1RN	37.9
Interleukin 10	IL10	1.6

M1 and M2 genes are referred to specific transcriptional profiles provided in references 28 and 29. The expression of the genes in bold letters was confirmed by qRT-PCR. nc : not changed. FC in microarray.
