# Supplementary material for: Orientia tsutsugamushi Stimulates an Original Gene Expression Program in Monocytes: Relationship with Gene Expression in Patients with Scrub Typhus
Source: PLoS Negl Trop Dis. 2011 May 17;5(5):e1028. doi: 10.1371/journal.pntd.0001028 (PMC3096591; doi:10.1371/journal.pntd.0001028)
Supplement: Table S5 — Apoptosis-related genes in O. tsutsugamushi-stimulated monocytes. (PDF) [file pntd.0001028.s007.pdf]

Table S5. Apoptosis-related genes in *O. tsutsugamushi*-stimulated monocytes

Gene function and full gene name	Gene symbol	GenBank ID	FC
**Apoptosis**			
adenosine A2a receptor	ADORA2A	NM_000675	13.4
apoptosis-inducing factor, mitochondrion-associated, 2	AIFM2	NM_032797	2.4
AXIN1 up-regulated 1	AXUD1	NM_033027	12,2
B-cell receptor-associated protein 29	BCAP29	NM_001008407	2.5
baculoviral IAP repeat-containing 4	BIRC4	NM_001167	2.3
CD40 molecule, TNF receptor superfamily member 5	CD40	NM_001250	6.0
catenin, beta like 1	CTNNBL1	NM_030877	2.4
damage-regulated autophagy modulator	DRAM	BC018435	5.2
eukaryotic translation initiation factor 2-alpha kinase 2	EIF2AK2	NM_002759	13.0
Fas (TNF receptor superfamily, member 6)	FAS	NM_000043	3.3
immediate early response 3	IER3	NM_003897	5.3
interleukin 19	IL19	NM_153758	35.9
interleukin 1, alpha	IL1A	NM_000575	9.0
interleukin 1, beta	IL1B	NM_000576	20.7
Janus kinase 2 (a protein tyrosine kinase)	JAK2	NM_004972	4.9
lipopolysaccharide-induced TNF factor	LITAF	NM_004862	2.2
nuclear factor of kappa light polypeptide gene enhancer in B-cells 1 (p105)	NFKB1	NM_003998	3.1
oncostatin M	OSM	NM_020530	4.2
pleckstrin homology-like domain, family A, member 2	PHLDA2	NM_003311	4.9
protein phosphatase 1, regulatory (inhibitor) subunit 15A	PPP1R15A	NM_014330	3.1
receptor (TNFRSF)-interacting serine-threonine kinase 1	RIPK1	NM_003804	3.2
serine/threonine kinase 3 (STE20 homolog, yeast)	STK3	NM_006281	2.5
TIA1 cytotoxic granule-associated RNA binding protein	TIA1	NM_022173	2.5
TIA1 cytotoxic granule-associated RNA binding protein	TIA1	NM_022037	2.6
tumor necrosis factor (TNF superfamily, member 2)	TNF	NM_000594	13.9
tumor necrosis factor, alpha-induced protein 3	TNFAIP3	NM_006290	4.6
tumor necrosis factor receptor superfamily, member 10a	TNFRSF10A	NM_003844	4.3
tumor necrosis factor receptor superfamily, member 10d, decoy with truncated death domain	TNFRSF10D	NM_003840	2.9
tumor necrosis factor receptor superfamily, member 18	TNFRSF18	NM_148901	3.6
tumor necrosis factor (ligand) superfamily, member 9	TNFSF9	NM_003811	8.1
tumor necrosis factor (ligand) superfamily, member 10	TNFSF10	NM_003810	78.7
tumor necrosis factor (ligand) superfamily, member 15	TNFSF15	NM_005118	10.4
ubiquitin-conjugating enzyme E2Z	UBE2Z	NM_023079	2.4
**Anti-apoptosis**			
annexin A1	ANXA1	NM_000700	2.6
baculoviral IAP repeat-containing 4	BIRC4	NM_001167	2.3
CASP8 and FADD-like apoptosis regulator	CFLAR	NM_003879	3.2
CASP8 and FADD-like apoptosis regulator	CFLAR	AF009616	5.8
immediate early response 3	IER3	NM_003897	5.3
interferon, alpha-inducible protein 6	IFI6	NM_022873	10.2
interleukin 1, alpha	IL1A	NM_000575	9.0
nuclear factor of kappa light polypeptide gene enhancer in B-cells 1 (p105)	NFKB1	NM_003998	3.1
v-rel reticuloendotheliosis viral oncogene homolog A, nuclear factor of kappa light polypeptide gene enhancer in B-cells 3, p65 (avian)	RELA	BC014095	3.1
serpin peptidase inhibitor, clade B (ovalbumin), member 2	SERPINB2	NM_002575	13.9
suppressor of cytokine signaling 3	SOCS3	NM_003955	11.7
sphingosine kinase 1	SPHK1	NM_021972	2.6
tumor necrosis factor (TNF superfamily, member 2)	TNF	NM_000594	13.9

tumor necrosis factor, alpha-induced protein 3	TNFAIP3	NM_006290	4.6
tumor necrosis factor receptor superfamily, member 10d, decoy with truncated death domain	TNFRSF10D	NM_003840	2.9
tumor necrosis factor receptor superfamily, member 18	TNFRSF18	NM_148901	3.6
**Induction of apoptosis**			
apoptosis-inducing factor, mitochondrion-associated, 2	AIFM2	NM_032797	2.4
BCL2-antagonist/killer 1	BAK1	NM_001188	2.3
caspase 10, apoptosis-related cysteine peptidase	CASP10	NM_032977	5.7
caspase 10, apoptosis-related cysteine peptidase	CASP10	NM_032974	3.3
CD70 molecule	CD70	NM_001252	3.8
Fas (TNF receptor superfamily, member 6)	FAS	NM_000043	3.3
interferon, beta 1, fibroblast	IFNB1	NM_002176	71.7
interleukin 19	IL19	NM_153758	35.9
inhibin, beta A (activin A, activin AB alpha polypeptide)	INHBA	AK001903	51.5
inhibin, beta A	INHBA	NM_002192	39.0
myxovirus (influenza virus) resistance 1, interferon-inducible protein p78 (mouse)	MX1	NM_002462	26.7
pleiomorphic adenoma gene-like 1	PLAGL1	NM_006718	1.9
phorbol-12-myristate-13-acetate-induced protein 1	PMAIP1	NM_021127	4.5
promyelocytic leukemia	PML	NM_002675	7.3
promyelocytic leukemia	PML	NM_033238	10.3
promyelocytic leukemia	PML	NM_033244	9.5
promyelocytic leukemia	PML	NM_033247	8.1
TIA1 cytotoxic granule-associated RNA binding protein	TIA1	NM_022173	2.5
TIA1 cytotoxic granule-associated RNA binding protein	TIA1	NM_022037	2.6
toll-like receptor 2	TLR2	NM_003264	3,1
tumor necrosis factor receptor superfamily, member 10a	TNFRSF10A	NM_003844	4.3
tumor necrosis factor receptor superfamily, member 9	TNFRSF9	NM_003811	5.8
tumor necrosis factor (ligand) superfamily, member 10	TNFSF10	NM_003810	78.7
**Regulation of apoptosis**			
BCL2-antagonist/killer 1	BAK1	NM_001188	2.3
BCL2-like 14 (apoptosis facilitator)	BCL2L14	NM_030766	20.6
B-cell translocation gene 1, anti-proliferative	BTG1	NM_001731	3.7
caspase 10, apoptosis-related cysteine peptidase	CASP10	NM_032977	5.7
caspase 10, apoptosis-related cysteine peptidase	CASP10	NM_032974	3.3
CASP8 and FADD-like apoptosis regulator	CFLAR	NM_003879	3.2
CASP8 and FADD-like apoptosis regulator	CFLAR	AF009616	5.8
Fas (TNF receptor superfamily, member 6)	FAS	NM_000043	3.3
interferon induced with helicase C domain 1	IFIH1	NM_022168	18.0
nucleotide-binding oligomerization domain containing 2	NOD2	NM_022162	2.0
TNF receptor-associated factor 1	TRAF1	NM_005658	6.7
