# Supplementary material for: Orientia tsutsugamushi Stimulates an Original Gene Expression Program in Monocytes: Relationship with Gene Expression in Patients with Scrub Typhus
Source: PLoS Negl Trop Dis. 2011 May 17;5(5):e1028. doi: 10.1371/journal.pntd.0001028 (PMC3096591; doi:10.1371/journal.pntd.0001028)
Supplement: Table S6 — List of the 65 transcripts specific for scrub typhus. (PDF) [file pntd.0001028.s008.pdf]

Table S6. List of the 65 transcripts specific for scrub typhus

down-modulated genes in scrub typhus				
BCL11A	CORO1B	FOSB	GAGE6	LDLRAP1
RAPGEF3	SLC22A18	Hs.552434	LOC653374	

up-regulated genes in scrub typhus				
ADD3	FAM107B	RASA2	UBE1L2	Hs.562182
ATP13A3	FBXO4	RKHD2	UGCGL1	Hs.250648
AYTL2	FLJ20160	RORA	UHMK1	LOC285513
C9orf77	MACF1	SLC25A32	ZBTB2	LOC642161
CBLB	NUP54	SRPK2	ZMYND11	LOC649801
CD3G	NXF5	SUMO2	ZNF326	LOC651633
CD8A	NXT2	SYNE2	ZNF644	LOC652282
CD8B1	PLEKHF1	SYTL2	Hs.561954	LOC653115
DLD	PPP2R5C[a]	TCEA1	Hs.573541	LOC653663
DLG1	PRKACB[a]	TCERG1	Hs.373705	LOC653675
DNTTIP2	PRPS1L1	TOPBP1	Hs.567392	

Down-modulated and up-regulated genes in scrub typhus were compared to murine typhus, malaria and dengue, and were identified by Welch ANOVA ($P < 0.05$, FDR < 5%) followed by Tukey HSD post hoc test. [a]Two isoforms of gene transcripts are included in the list.
